# Mendelian randomization and clinical trial evidence supports TYK2 inhibition as a therapeutic target for autoimmune diseases

**DOI:** 10.1016/j.ebiom.2023.104488

**Published:** 2023-02-24

**Authors:** Shuai Yuan, Lijuan Wang, Han Zhang, Fengzhe Xu, Xuan Zhou, Lili Yu, Jing Sun, Jie Chen, Haochao Ying, Xiaolin Xu, Yongfu Yu, Athina Spiliopoulou, Xia Shen, Jim Wilson, Dipender Gill, Evropi Theodoratou, Susanna C. Larsson, Xue Li

**Affiliations:** a School of Public Health and the Second Affiliated Hospital, Zhejiang University School of Medicine, Hangzhou, China; b Unit of Cardiovascular and Nutritional Epidemiology, Institute of Environmental Medicine, Karolinska Institutet, Stockholm, Sweden; c Centre for Global Health Research, Usher Institute, University of Edinburgh, Edinburgh, UK; d College of Public Health, Zhengzhou University, Zhengzhou, China; e Key Laboratory of Growth Regulation and Translational Research of Zhejiang Province, School of Life Sciences, Westlake University, Hangzhou, China; f Department of Biostatistics, School of Public Health, and The Key Laboratory of Public Health Safety of Ministry of Education, Fudan University, Shanghai, China; g Centre for Public Health, Usher Institute, University of Edinburgh, Edinburgh, UK; h Center for Intelligent Medicine Research, Greater Bay Area Institute of Precision Medicine (Guangzhou), Fudan University, Guangzhou, China; i State Key Laboratory of Genetic Engineering, School of Life Sciences, Fudan University, Shanghai, China; j Department of Epidemiology and Biostatistics, School of Public Health, Imperial College London, London, UK; k Medical Research Council Biostatistics Unit, Cambridge Institute of Public Health, Cambridge, UK; l Cancer Research UK Edinburgh Centre, Medical Research Council Institute of Genetics and Cancer, University of Edinburgh, Edinburgh, UK; m Unit of Medical Epidemiology, Department of Surgical Sciences, Uppsala University, Uppsala, Sweden

**Keywords:** Autoimmune disease, Mendelian randomization, Colocalization, Drug development, TYK2

## Abstract

**Background:**

To explore the associations of genetically proxied TYK2 inhibition with a wide range of disease outcomes and biomarkers to identify therapeutic repurposing opportunities, adverse effects, and biomarkers of efficacy.

**Methods:**

The loss-of-function missense variant rs34536443 in *TYK2* gene was used as a genetic instrument to proxy the effect of TYK2 inhibition. A phenome-wide Mendelian randomization (MR) study was conducted to explore the associations of genetically-proxied *TYK2* inhibition with 1473 disease outcomes in UK Biobank (N = 339,197). Identified associations were examined for replication in FinnGen (N = 260,405). We further performed tissue-specific gene expression MR, colocalization analyses, and MR with 247 blood biomarkers. A systematic review of randomized controlled trials (RCTs) on TYK2 inhibitor was performed to complement the genetic evidence.

**Findings:**

PheWAS-MR found that genetically-proxied TYK2 inhibition was associated with lower risk of a wide range of autoimmune diseases. The associations with hypothyroidism and psoriasis were confirmed in MR analysis of tissue-specific *TYK2* gene expression and the associations with systemic lupus erythematosus, psoriasis, and rheumatoid arthritis were observed in colocalization analysis. There were nominal associations of genetically-proxied TYK2 inhibition with increased risk of prostate and breast cancer but not in tissue-specific expression MR or colocalization analyses. Thirty-seven blood biomarkers were associated with the TYK2 loss-of-function mutation. Evidence from RCTs confirmed the effectiveness of TYK2 inhibitors on plaque psoriasis and reported several adverse effects.

**Interpretation:**

This study supports TYK2 inhibitor as a potential treatment for psoriasis and several other autoimmune diseases. Increased pharmacovigilance is warranted in relation to the potential adverse effects.

**Funding:**

None.

**Research in context**

**Evidence before this study**

Deucravacitinib is a selective inhibitor of tyrosine kinase 2 (TYK2) and has been approved to treat moderate-to-severe plaque psoriasis. TYK2 belongs to the Janus kinase family that exerts effects on a wide range of inflammatory disorders. Thus, TYK2 inhibitors may have the potential in the treatment for autoimmune diseases. However, relatively few clinical trials on autoimmune diseases except psoriasis hinder the assessment of the effectiveness of TYK2 inhibitor treatment on autoimmune diseases. In addition, Janus kinase inhibitors have been associated with increased risk of serious heart-related events and certain cancers, which similarly raises concerns on their safety. No studies have been conducted to systematically explore the possible adverse effects of TYK2 inhibitor.

**Added value of this study**

This comprehensive study found evidence supporting the efficacy of TYK2 inhibitors for psoriasis and its related disorders. There were Mendelian randomization associations of the *TYK2* loss-of-function variant with hypothyroidism, inflammatory bowel disease, primary biliary cirrhosis, and type 1 diabetes. Although only a few clinical trials supported that TYK2 inhibitors appeared to improve disease activity among patients with ulcerative colitis, alopecia areata, atopic dermatitis, or active non-segmental vitiligo, these findings need to be confirmed in larger studies, especially for ulcerative colitis, for which there was conflicting evidence in previous trials. The study identified several potential adverse effects of TYK2 inhibitors, including headache, upper respiratory tract infection, nausea, diarrheal, increased circulating levels of creatinine and liver enzymes, and risk of certain malignant neoplasms, such prostate and breast cancer.

**Implications of all the available evidence**

TYK2 inhibitors may be used to treat psoriasis and possibly other autoimmune diseases, like hypothyroidism, inflammatory bowel disease, primary biliary cirrhosis, and type 1 diabetes. The side effects of TYK2 inhibitors should be assessed, especially on prostate and breast cancer.

## Introduction

Deucravacitinib, a selective inhibitor of tyrosine kinase 2 (TYK2), has been approved to treat moderate-to-severe plaque psoriasis.^1^^,^^2^ Given that TYK2 belongs to the Janus kinase (JAK) family that exerts effects on a wide range of inflammatory disorders, TYK2 inhibitors may have the potential in the treatment for other autoimmune diseases, such as inflammatory bowel disease,^3^ rheumatoid arthritis,^4^ and type 1 diabetes.^5^ However, relatively few clinical trials on these outcomes hinder the assessment of the effectiveness of TYK2 inhibitor treatment on autoimmune diseases beyond plaque psoriasis.^6^^,^^7^ In addition, three JAK inhibitors have been recently associated with increased risk of serious heart-related events and certain cancers,^8^ which similarly raises concerns on their safety. A recent Mendelian randomization (MR) study observed positive associations of a *TYK2* loss-of-function mutation that mimic TYK2 inhibition with increased risk of lung cancer, non-Hodgkin lymphoma, and possibly prostate cancer.^9^ However, no studies have been conducted to systematically explore the possible adverse effects of inhibiting this drug target.

In the absence of long-term randomized controlled trials (RCTs) investigating TYK2 inhibition, MR analysis can be used to assess the effectiveness, repurposing potential, and safety of TYK2 inhibition by utilizing genetic variants in the *TYK2* gene that reduce its function as instrumental variables for life-time TYK2 inhibition.^10^^,^^11^ Resembling the RCT study design, the MR approach naturally randomizes participants into groups based on genetically predicted drug target perturbation, and thus diminishes confounding effects from environmental factors since genetic variants are randomly assorted at conception. In addition, this approach can minimize reverse causality as the onset and progression of disease cannot modify the germline genotype. Here, we performed an MR investigation to comprehensively explore disease and biomarker phenotypes associated with a *TYK2* loss-of-function genetic variant. To strengthen and complement the MR results, we performed a review of RCTs on TYK2 inhibition to investigate the effectiveness and safety of this drug.

## Methods

### Study design and ethics permit

**Fig. 1:**
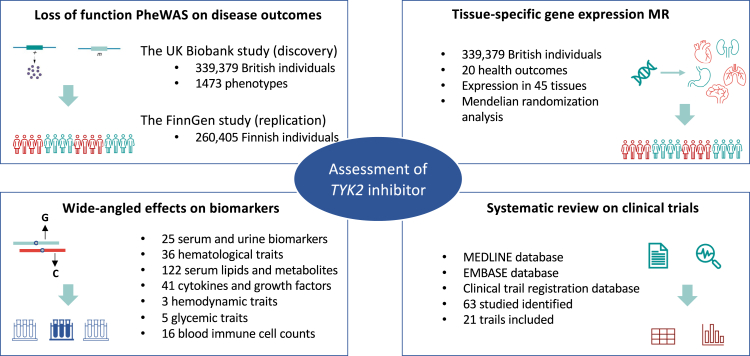
Study design overview.

The study design overview is presented in Fig. 1. We firstly performed a phenome-wide association study (PheWAS) to comprehensively examine the associations of the loss-of-function mutation in the *TYK2* gene with disease outcomes in the UK Biobank study. We then conducted a Mendelian randomization (MR) analysis in the FinnGen study with the aim of replicating the identified PheWAS associations. To further investigate the evidence for causality, tissue-specific gene expression and colocalization analyses were performed to examine the associations between *TYK2* gene expression on certain tissue and risk of diseases highlighted in PheWAS-MR. We also explored the MR associations of TYK2 with a wide range of biomarkers, including haematological, biochemical, metabolomic, inflammatory, and immunological traits in data from phenotype-specific genetic consortia and performed mediation analysis of pathophysiological mechanisms pathways from TYK2 inhibition to disease outcomes. Finally, we collected data on published RCTs on TYK2 inhibition to complement the genetic evidence of possible clinical effects. UK Biobank received ethical permits from the Northwest Multi-centre Research Ethics Committee, the National Information Governance Board for Health and Social Care in England and Wales, and the Community Health Index Advisory Group in Scotland. All participants provided written informed consent.

### Phenome-wide association study of TYK2 mutation in the UK Biobank

PheWAS analysis of the loss-of-function mutation in *TYK2* gene was performed in the UK Biobank study, an ongoing cohort study collecting phenotypic and genetic data from over 500,000 individuals since its initiation in 2006–2010. After removal of participants of other descents to minimize population bias, the current study was based on data from 339,197 (182,072 females and 157,125 males) unrelated White British individuals. Health outcomes were defined by using the PheCODE schema with diagnostic codes (10,750 unique ICD-10 codes and 3113 ICD-9 codes) from national medical records (inpatient hospital episode records, cancer registry, and death registry).^12^ The PheCODE system provides a scheme to automatically exclude patients that have similar or potentially overlapping disease states from the corresponding control group. We used the International Classification of Diseases (ICD) versions 9 and 10 to identify cases in the medical records, with both incident and prevalent cases included. A map matching ICD-9 and -10 codes to phecodes was used, as previously described (https://phewascatalog.org/phecodes_icd10).^13^ Detailed information on genotyping and quality control is described in our previous studies.^14^^,^^15^

### Validating PheWAS associations in the FinnGen Biobank

For phenotypes reaching statistical significance after FDR correction in the original PheWAS analysis, we further examined associations with the missense variant rs34536443 of the *TYK2* gene in the FinnGen (N = 260,405) study. The FinnGen study is a growing project combining germline genotype data from Finnish biobanks and health record data on clinically defined outcomes from Finnish health registries in up to 260,405 individuals.^16^ We performed an MR study in R6 release of the FinnGen study to investigate replication of the identified PheWAS associations (https://finngen.gitbook.io/documentation/).

### Tissue-specific *TYK2* expression and related disease outcomes

We carried out tissue-specific expression analysis of *TYK2* gene to examine the associations between gene expression levels and related health outcomes identified from the loss-of-function PheWAS analysis, using the PrediXcan software.^17^ The analysis was based on the same sample from UK Biobank as for PheWAS. PrediXcan first uses reference transcriptome datasets to train additive models of gene expression levels, providing the effect sizes of single nucleotide polymorphisms (SNPs) on gene expression (i.e., prediction weights). We used expression weights from 45 tissues in the genotype-tissue expression (GTEx) database^18^ as reference panels and the prepackaged expression weights can be downloaded directly from the PredictDB data repository. Then, PrediXcan imputed the genetic component of expression by integrating genotype data from large-scale genome-wide association studies (GWASs) and prediction weights from the training sets while accounting for linkage disequilibrium among SNPs. Last, PrediXcan correlates the genetically predicted gene expression with the disease phenotypes using logistic regression methods. We applied a Benjamini-Hochberg correction to account for multiple testing in each tissue and associations with FDR <0.05 were considered as statistically significant.

### Colocalization analysis of *TYK2* gene tissue-specific expression with disease outcomes

To further investigate causality of observed MR associations, we performed colocalization analysis of *TYK2* gene tissue-specific expression (eQTL) with risk of common autoimmune diseases (including psoriasis,^19^ rheumatoid arthritis,^20^ inflammatory bowel disease,^21^ systemic lupus erythematosus,^22^ multiple sclerosis,^23^ and type 1 diabetes^24^) and related cancers (prostate^25^ and breast^26^ cancers) with publicly available genome-wide association data. This colocalization analysis can infer whether TYK2 expression and the risk of above autoimmune disease are affected by the same genetic variant. SNPs in *TYK2* gene region ±1000 kb were used as instruments. Data on *TYK2* expression in different tissues were obtained from the GTEx database.^18^ We additionally used data on *TYK2* expression in whole blood from the eQTLGen dataset.^27^ Summary-level data on the associations of used SNPs with the outcomes were obtained from above cited GWASs. We used coloc method to obtain posterior probability for 5 hypotheses (H0–H4) in a Bayesian framework.^28^ PP.H4 <80% of the colocalization analysis (H4) indicates absence of strong support for a shared causal variant affecting gene expression and disease risk. We also applied the Sum of Single Effects (SuSiE) colocalization method that allows multiple signals to be distinguished to filter out linkage disequilibrium-contaminated associations.^29^ The analyses were performed using the default priors (p1 = 1 × 10^−4^, p2 = 1 × 10^−4^, and p12 = 1 × 10^−5^). *F* statistics were estimated for each eQTL signal across tissues. The analyses were performed using coloc 5.1 package in R 3.5.1.^30^

### Biomarker-wide association and mediation analyses

We obtained association estimates of the loss-of-function mutation of *TYK2* gene with the following biomarkers: (i) 25 serum and urine biomarkers available in the biochemistry panel of the UK Biobank (353,579 individuals); (ii) 36 haematological traits with data derived from the summary statistics of the study by Astle et al. (173,480 European individuals)^31^; (iii) 122 nuclear magnetic resonance-measured serum lipids and metabolites with data derived from the publicly available summary statistics provided by Kettunen et al. (24,925 individuals of European ancestry)^32^; (iv) circulating levels of 41 cytokines and growth factors with data derived from the publicly available summary statistics by Ahola-Olli et al. (8293 individuals of Finnish ancestry)^33^; (v) 3 hemodynamic traits that were available in the UK Biobank (408,228 individuals); (vi) 5 glycaemic traits made publicly available from a series of analyses from the MAGIC Consortium (up to 133,010 individuals)^34^; and (vii) 16 blood immune cell counts derived from the summary statistics made publicly available by Orrù V et al. (3757 individuals).^35^ The data sources for these studies are described in Supplementary Table S1.

To uncover pathophysiological mechanisms pathways from TYK2 inhibitor to autoimmune disease, we performed causal mediation analysis (CMA) for certain identified biomarkers using the mediation R package^36^ in the UK Biobank study. We obtained an average causal mediation effect (ACME) that is transmitted via mediator to the outcome and an average direct effect that explained by the exposure as well as the proportion of explained variance by the mediator from this analysis.^36^

### Systematic review of clinical drug trials on TYK2 inhibitors

We conducted a systematic review on clinical trials of TYK2 inhibitors by searching corresponding studies in three databases: MEDLINE, EMBASE, and the clinical trials registration database, published until March 30th, 2022. Full search strategies are shown in Supplementary Table S2. Studies that were not RCT or not based on humans, were excluded. Information on the first author, year of study, National Clinical Trial number, characteristics of included patients, sample size, intervention, phase of trial, status of trial, assessment of efficacy and adverse effects were extracted. The literature search, review process, and data extraction were done in parallel by two authors (S.Y and X.Z.).

### Statistical analysis

The associations of rs34536443 with disease outcomes was estimated by logistic regression, and levels of biomarkers by linear regression. The PheWAS compared the risk of outcomes between individual carrying and not carrying rare *TYK2* loss-of-function mutation, and the logistic regression model was adjusted for age, sex, body mass index, assessment centre, and first 10 principal genetic components. MR analysis in FinnGen and tissue-specific gene expression MR analysis was based on logistic regression with an additive [per minor (C) allele] genetic model adjusting for age, sex, 10 genetic principal components, and genotyping batch in FinnGen, and adjusting for age, sex, assessment centre, and first 10 principal genetic components in tissue-specific gene expression MR. Covariates adjusted in biomarker-wide MR analysis are presented in Supplementary Table S1. We applied a Benjamini-Hochberg correction to account for multiple testing in each analysis with FDR <0.05 were considered as statistically significant.

### Role of funding source

The funding sources had no role in the design of this study and did not have any role in the data collection, data analyses, interpretation, writing of report, or decision to submit results.

## Results

### PheWAS identified 19 disease outcomes associated with *TYK2* inhibition in UK Biobank

**Fig. 2:**
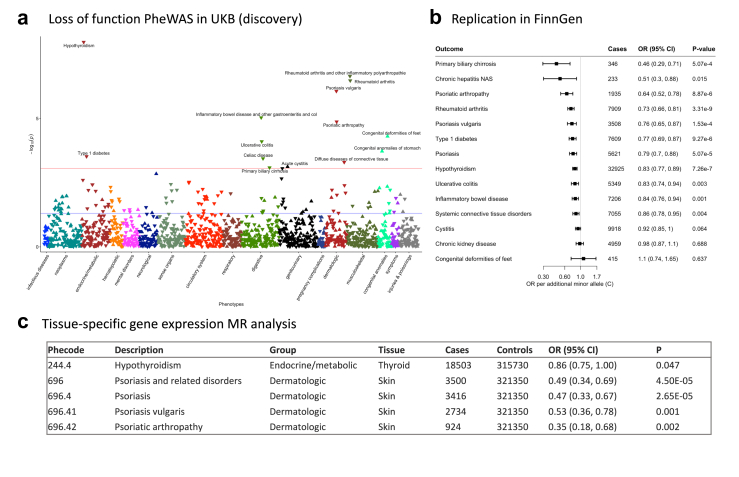
Summary of results from Mendelian randomization (MR) analysis on disease outcomes. a, MR-PheWAS analysis of the associations between *TYK2* loss-of-function mutation and health outcomes. b, MR analysis of the health effects of *TYK2* inhibition on disease outcomes. c, Tissue-specific gene expression analysis for validating the associations between *TYK2* expression and health outcomes. CI, confidence interval; OR, odds ratio; UKB, UK Biobank.

The characteristics of 339,197 individual in UK Biobank are displayed in Supplementary Table S3. We defined 1473 phenotypes using the PheCODE schema after removing outcomes with less than 200 cases in UK Biobank (Supplementary Table S4). The MR-PheWAS analysis identified 119 outcomes nominally associated with the loss-of-function mutation of *TYK2* (Supplementary Table S5), and sixteen outcomes showed significant associations after multiple-testing correction (Fig. 2a and Table 1). The mappings of ICD codes to these health outcomes are shown in Supplementary Table S6. In detail, the *TYK2* loss-of-function mutation was associated with decreased risk of hypothyroidism, psoriasis and its related disorders, psoriasis vulgaris, rheumatoid arthritis and other inflammatory polyarthropathies, psoriatic arthropathy, chronic hepatitis, ulcerative colitis, inflammatory bowel disease and other gastroenteritis and colitis, celiac disease, noninfectious gastroenteritis, type 1 diabetes, disorders of eye, and increased risk of congenital deformities of feet and congenital anomalies of stomach (Table 1).

**Table 1:** Outcomes associated with the *TYK2* loss-of-function mutation in MR-PheWAS analysis in the UK Biobank.

Phecode	Phenotype	Group	Cases	Controls	Beta	SE	OR	*P*
244.4	Hypothyroidism	endocrine/metabolic	18,503	315,717	−0.18	0.03	0.84	6.23E-10
696.4	Psoriasis	dermatologic	2589	301,676	−0.46	0.08	0.63	4.11E-08
246	Other disorders of thyroid	endocrine/metabolic	21,850	315,717	−0.14	0.03	0.87	5.26E-08
696.41	Psoriasis vulgaris	dermatologic	2751	301,676	−0.39	0.08	0.67	5.42E-07
714.1	Rheumatoid arthritis	musculoskeletal	5906	304,719	−0.24	0.05	0.79	2.79E-06
714	Rheumatoid arthritis and other inflammatory polyarthropathies	musculoskeletal	30,060	304,719	−0.10	0.02	0.90	6.26E-06
696.42	Psoriatic arthropathy	dermatologic	929	301,676	−0.66	0.15	0.52	1.59E-05
755.1	Congenital deformities of feet	congenital anomalies	273	336,622	0.65	0.16	1.91	4.89E-05
70.4	Chronic hepatitis	infectious diseases	341	330,659	−1.27	0.33	0.28	1.50E-04
750.15	Congenital anomalies of stomach	congenital anomalies	73	335,451	1.00	0.27	2.72	1.89E-04
555.2	Ulcerative colitis	digestive	3269	251,815	−0.25	0.07	0.78	2.83E-04
555	Inflammatory bowel disease and other gastroenteritis and colitis	digestive	19,792	251,815	−0.10	0.03	0.91	2.99E-04
557.1	Celiac disease	digestive	2185	251,815	−0.31	0.08	0.74	3.08E-04
558	Non-infectious gastroenteritis	digestive	19,875	251,815	−0.10	0.03	0.91	3.31E-04
250.1	Type 1 diabetes	endocrine/metabolic	2862	311,499	−0.26	0.07	0.77	3.68E-04
379	Other disorders of eye	sense organs	57,586	280,543	−0.06	0.02	0.94	4.77E-04

CI, confidence interval; OR, odds ratio; SE, standard error.

The risk of outcomes was calculated by comparing odds between individual carrying and not carrying the rare *TYK2* loss-of-function mutation.

### Health effects of *TYK2* inhibition on autoimmune diseases were successfully replicated in FinnGen Biobank

The results showed that eleven related disease outcomes were successfully replicated in MR analysis in FinnGen (Fig. 2b and Supplementary Table S7). Per minor (C) allele increase of rs34536443, the odds ratio (OR) was 0.46 (95% confidence interval [CI] 0.29, 0.71) for primary biliary cirrhosis, 0.51 (95% CI 0.30, 0.88) for chronic hepatitis, 0.64 (95% CI 0.52, 0.78) for psoriatic arthropathy, 0.73 (95% CI 0.66, 0.81) for rheumatoid arthritis, 0.76 (95% CI 0.65, 0.87) for psoriasis vulgaris, 0.77 (95% CI 0.69, 0.87) for type 1 diabetes, 0.79 (95% CI 0.70, 0.88) for psoriasis, 0.83 (95% CI 0.77, 0.89) for hypothyroidism, 0.83 (95% CI 0.74, 0.94) for ulcerative colitis, 0.84 (95% CI 0.76, 0.94) for inflammatory bowel disease, and 0.86 (95% CI 0.78, 0.95) for systemic connective tissue disorders. No associations were observed between rs34536443 and cystitis, chronic kidney disease, and congenital deformities of feet. No data were available for congenital anomalies of stomach or celiac disease in FinnGen.

### Tissue-specific expression analyses verified the associations between *TYK2* expression and disease outcomes across multi-tissues

Tissue-specific gene expression analyses verified that the loss-of-function mutation of rs34536443 was associated with differential expression of *TYK2* in multiple tissues, particularly whole blood, visceral adipose, colon, skin, testis (Supplementary Fig.S1). We observed several associations between *TYK2* expression and disease outcomes in tissues where disease occurs. Specifically, there were inverse associations of lower *TYK2* expression in thyroid with reduced risk of hypothyroidism (OR, 0.86; 95% CI 0.75, 1.00), in skin with psoriasis and its related disorders (OR, 0.49; 95% CI 0.34, 0.69), psoriasis (OR, 0.47; 95% CI 0.33, 0.67), psoriasis vulgaris (OR, 0.53; 95% CI 0.36, 0.78), and psoriatic arthropathy (OR, 0.35; 95% CI 0.18, 0.68) (Fig. 2c). Differential gene expression in other tissues also showed associations with diseases in MR-PheWAS where corresponding pathophysiology does not typically manifest (Supplementary Table S8).

### Malignant neoplasm associated with genetically proxied TYK2 inhibition

Even though there were no significant associations between genetically proxied TYK2 inhibition and risk of different cancers after correction for multiple comparison, three malignant neoplasms, including malignant neoplasm of prostate, male genital organs, and breast showed consistent suggestive positive associations with the *TYK2* loss-of-function mutation in UK Biobank and FinnGen (Supplementary Table S9). Tissue-specific expression analyses showed reduced expression of *TYK2* in breast tissue was associated with increased risk of breast cancer (OR, 1.21; 95% CI 1.02, 1.43), but there were no associations with cancers of the prostate or male genital organs at corresponding tissues. Colocalization analysis observed no associations of *TYK2* expression with prostate or breast cancer in any tissues (PP <50%).

### Colocalization analysis of tissue specific *TYK2* expression with disease outcomes

In total, 18 of 49 tissues had *TYK2* eQTL signals at the genome-wide significant level (P < 5 × 10^−8^) and the *F* statistics of the signals ranged from 16 to 67 across tissues (Supplementary Table S10). Twelve associations of *TYK2* gene expression with 6 autoimmune diseases in 8 tissues were identified in colocalization analysis (PP>80%). Specifically, *TYK2* gene expression showed colocalized associations with systemic lupus erythematosus in lower leg skin (PP = 100%), whole blood (PP = 99%), artery tibial (PP = 98%), adrenal gland (PP = 98%), and stomach (PP = 91%), psoriasis in whole blood (PP = 99%), ulcerative colitis (PP = 97%) and inflammatory bowel disease (PP = 93%) in brain hypothalamus, Crohn's Disease in artery tibial (PP = 97%), oesophagus muscularis (PP = 92%), and oesophagus gastroesophageal junction (PP = 87%), and rheumatoid arthritis in whole blood (PP = 88%). There were two hits prioritized by SuSiE analysis shared between *TYK2* expression and above outcomes in several tissues, and additionally type 1 diabetes in visceral adipose and lung (Supplementary Table S11).

### Effects of genetically proxied TYK2 inhibition on multiple disease-related biomarkers

**Fig. 3:**
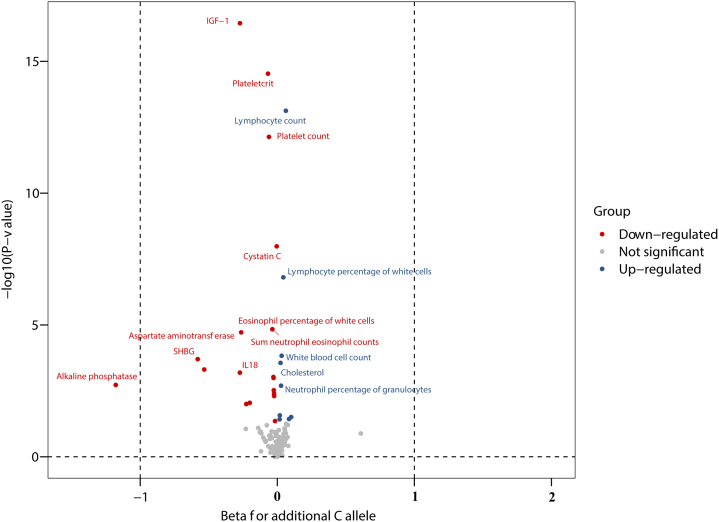
Biomarkers associated with additional minor (C) allele of rs34536443 in TYK2 gene regression. CI, confidence interval. The associations survived after multiple testing were labelled in the volcano plot.

To gain additional insights into the relationships between TYK2 function and subclinical endophenotypes relevant to human diseases, we explored associations between the *TYK2* loss-of-function variant and eight categories of 247 biomarkers derived from different sources, as detailed in Supplementary Table S12. The results, along with the number of individuals examined in each analysis are presented in Supplementary Table S12. Forty-four out of 247 biomarkers were nominally associated with rs34536443 (Supplementary Table S12). The associations for 37 of 44 biomarkers survived after multiple testing correction, mostly belonging to blood immune cell, haematological traits, and serum/urine biochemistry parameters (Fig. 3 and Supplementary Table S12). For each additional minor (C) allele of rs34536443, the levels of rheumatoid factor decreased by −1.21 (95% CI -1.98, −0.44) and the count of lymphocyte increased by 0.32 (95% 0.18, 0.47) (Fig. 3).

We performed the CMA for Cystatin C, insulin-like growth factor 1, sex hormone binding globulin, and interleukin 18 (Supplementary Table S13). We observed Cystatin C mediated the association of TYK2 mutations with hypothyroidism (*P* for ACME <0.001), rheumatoid arthritis (*P* for ACME = 0.02), ulcerative colitis (*P* for ACME = 0.02), chronic hepatitis (*P* for ACME <0.001), type 1 diabetes (*P* for ACME <0.001), Celiac disease (*P* for ACME <0.001), and diffuse diseases of connective tissue (*P* for ACME <0.001). Two mediation effects were observed for insulin-like growth factor 1 on the associations for hypothyroidism (*P* for ACME <0.001) and Celiac disease (*P* for ACME <0.001). There were no mediations observed for other biomarkers in the association between TYK2 mutations and observed outcomes in the UK Biobank (Supplementary Table S13).

### Review of RCTs on TYK2 inhibitors

A total of 23 published trials were identified in MEDLINE and 110 in EMBASE. After merging papers from two databases and removal of duplicates, 65 studies were included for screening. After title, abstract, and full-text screening, 19 studies were included. Along with 3 additional trials with published results identified in clinicaltrail.gov registration database, we included 21 RCTs on TYK2 inhibitors in this systematic review (Supplementary Fig.S2). The characteristics of 21 included RCTs are presented in Supplementary Table S14. In brief, these RCTs focused on examining the treatment effectiveness of TYK2 inhibitors on plaque psoriasis and a few studied ulcerative colitis, alopecia areata, systemic lupus erythematosus, atopic dermatitis, and active non-segmental vitiligo. These RCTs included both women and men with a wide range of age and the sample size ranged from 30 to 66.

Fifteen studies reported data on effectiveness of TYK2 inhibitors treatment on the target disease (Table 2). For plaque psoriasis, all studies (n = 7) found improved disease activity measured by the Psoriasis Area and Severity Index in the intervention groups with different doses compared to the control group. Likewise, disease activity improved in the intervention compared to control group among patients with psoriatic arthritis (n = 2), alopecia areata (n = 2), atopic dermatitis (n = 1), or active non-segmental vitiligo (n = 1) although a few studies were conducted in these diseases. TYK2 inhibitors improved certain clinical measures of ulcerative colitis severity, like improved modified Mayo endoscopic and Mayo rectal bleeding sub-score in the intervention group; however, there was no strong evidence of effect on clinical remission. Possible adverse effects of TYK2 inhibitors identified are presented in Supplementary Table S14. The most common complaints among individuals with TYK2 inhibitors treatment are headache, upper respiratory tract infection, nausea, diarrhoea, and increased circulating levels of creatinine and liver enzymes. Two RCTs reported cancer as the possible adverse effect of TYK2 inhibitor (Supplementary Table S14). Except for the above RCTs, there were some additional trails registered with the aim of exploring the effectiveness of TYK2 inhibitors on inflammatory bowel disease and systemic lupus erythematosus as well as assessing safety (Supplementary Table S15).

**Table 2:** Effectiveness assessment of TYK2 inhibitor in randomized controlled trails.

Study	NCT number	Drug	Condition	Clinical endpoint	Intervention	N	Estimation Parameter	Estimated Value	P value
Banfield 2018	NCT02310750	PF-06700841	Plaque psoriasis	Change from baseline in PASI score after 4 weeks	PBO 30 mg QD 100 mg QD	9 14 7	Maximal mean percent change	Ref −67.92% −96.31%	– – –
Papp 2018	NCT02931838	BMS-986165	Plaque psoriasis	75% or greater reduction from baseline in PASI score at week 12 (primary) 50% or greater reduction from baseline in PASI score at week 12 90% or greater reduction from baseline in PASI score at week 12 100% reduction from baseline in PASI score at week 12 sPGA score of 0 or 1 DLQI score of 0 or 1	PBO 3 mg QOD 3 mg QD 3 mg BID 6 mg BID 12 mg QD PBO 3 mg QOD 3 mg QD 3 mg BID 6 mg BID 12 mg QD PBO 3 mg QOD 3 mg QD 3 mg BID 6 mg BID 12 mg QD PBO 3 mg QOD 3 mg QD 3 mg BID 6 mg BID 12 mg QD PBO 3 mg QOD 3 mg QD 3 mg BID 6 mg BID 12 mg QD PBO 3 mg QOD 3 mg QD 3 mg BID 6 mg BID 12 mg QD	45 44 44 45 45 44 45 44 44 45 45 44 45 44 44 45 45 44 45 44 44 45 45 44 45 44 44 45 45 44 45 44 44 45 45 44	Proportion Percentage difference Percentage difference Percentage difference Percentage difference Percentage difference	7% 9% 39% 69% 67% 75% Ref 12 (−8, 32) 37 (18, 56) 60 (41, 75) 47 (29, 65) 58 (41, 74) Ref 5 (−16, 25) 14 (−7, 33) 42 (21, 60) 42 (21, 60) 41 (20, 58) Ref 2 (−18, 23) – 9 (−13, 30) 18 (−4, 38) 25 (4, 44) Ref 14 (−7, 33) 32 (11, 50) 69 (51, 83) 58 (38, 74) 68 (50, 82) Ref 12 (−2, 26) 12 (−2, 26) 38 (20, 54) 56 (38, 71) 59 (41, 74)	Ref 0.49 <0.001 <0.001 <0.001 <0.001 – – – – – – – – – – – – – – – – – – Ref – – – – – Ref – – – – –
Forman 2020	NCT02969018	PF-06700841	Plaque psoriasis	Change from baseline in PASI score at week 12 Proportion of patients achieving 75% reduction from baseline PASI at week 12 Proportion of patients achieving 90% reduction from baseline PASI at week 12	PBO 30 mg QD PBO 30 mg QD PBO 30 mg QD	23 29 23 29 23 29	LS mean difference Proportion Proportion	Ref −17.3 (−20.0, −14.6) Ref 86.20% Ref 51.70%	Ref <0.0001 – – – –
Sandbron 2020	NCT02818686	TD-1473	Ulcerative colitis	Rates of clinical response and endoscopic response on day 28 Rates of modified Mayo endoscopic and Mayo rectal bleeding sub-score improvement from baseline at day 28 Change in Robarts Histopathology Index from baseline to day 28	PBO 20 mg QD 80 mg QD 270 mg QD PBO 20 mg QD 80 mg QD 270 mg QD PBO 20 mg QD 80 mg QD 270 mg QD	9 10 10 11 9 10 10 11 9 10 10 11	Rate Rate mean	11% (clinical) 0% (endoscopic) 20% (clinical) 20% (endoscopic) 20% (clinical) 20% (endoscopic) 55% (clinical) 9% (endoscopic) 0% (endoscopy) 44% (rectal bleeding) 20% (endoscopy) 30% (rectal bleeding) 30% (endoscopy) 70% (rectal bleeding) 18% (endoscopy) 73% (rectal bleeding) −2 −4.5 1.8 −5.3	– – – – – – – – – – – –
Armstrong 2021	NCT03624127	BMS-986165	Plaque psoriasis	PASI 75 response versus placebo at Week 16 sPGA 0/1 response versus placebo at Week 16	PBO 6 mg QD Apremilast 30 mg BID PBO 6 mg QD Apremilast 30 mg BID	165 322 168 165 322 168	Proportion Proportion	12.70% 58.70% 35.10% 7.20% 53.60% 32.10%	Ref ^1^ <0.0001 Ref ^2^ Ref ^1^ <0.0001 Ref ^2^
Tehliran 2021	NCT03210961	PF-06826647	Plaque psoriasis	Change in PASI score at day 28	PBO 100 mg QD 400 mg QD	14 11 15	LS mean difference	Ref −3.49 (−9.48, 2.50) −13.05 (−18.76, −7.35)	Ref 0.33 0.00077
King 2021	NCT02974868	PF-06700841	Alopecia areata	Change from baseline in SALT score at week 24 Proportion of patients achieving 30% improvement in SALT score at week 24	PBO 60 mg QD for 4 ws 30 mg QD for 20 ws PBO 60 mg QD for 4 ws 30 mg QD for 20 ws	47 47 47 47	LS mean difference Proportion	Ref 49.2 (36.6, 61.7) – 64% (51%, 75%)	Ref <0.001 – –
Mease 2021	NCT03963401	PF-06700841	Psoriatic arthritis	ACR-20 response at week 16	PBO 10 mg QD 30 mg QD 60 mg QD	67 31 60 59	Proportion	29% 20% 40% 44%	Ref >0.05 <0.05 <0.05
Danese 2022	NCT03934216	BMS-986165	Ulcerative colitis	Clinical remission evaluated by modified Mayo score at week 12	PBO 6 mg BID	43 88	Proportion	16.30% 14.80%	Ref 0.59
Mease 2022	NCT03881059	BMS-986165	Psoriatic arthritis	ACR-20 response at week 16 Change from baseline in HAQ-DI score at week 16 PASI-75 response at week 16 Change from baseline in SF-36 PCS at week 16	PBO 6 mg QD 12 mg QD PBO 6 mg QD 12 mg QD PBO 6 mg QD 12 mg QD PBO 6 mg QD 12 mg QD	66 70 67 66 70 67 66 70 67 66 70 67	Adjusted OR Mean difference Adjusted OR Mean difference	Ref 2.4 (1.2, 4.8) 3.6 (1.8, 7.4) Ref −0.3 (−0.4, −0.1) −0.3 (−0.5, −0.1) Ref 2.9 (1.3, 6.7) 5.8 (2.4, 13.8) Ref 3.3 (0.9, 5.7) 3.5 (1.1, 5.9)	Ref 0.0134 0.0004 Ref 0.002 0.0008 Ref 0.0136 <0.0001 Ref 0.0062 0.0042
Thaci 2022	NCT02931838	BMS-986165	Plaque Psoriasis	Percentages of patients who achieved absolute PASI ≤ 1, absolute PASI ≤ 3, absolute PASI ≤ 5 Percentages of patients who achieved BSA ≤ 1% and BSA ≤ 3% Percentages of patients who achieved ≥ 75% improvement in sPGA × BSA	PBO 3 mg BID 6 mg BID 12 mg QD PBO 3 mg BID 6 mg BID 12 mg QD PBO 3 mg BID 6 mg BID 12 mg QD	45 45 45 44 45 45 45 44 45 45 45 44	Proportion Proportion Proportion	0%, 2.2%, 8.9% 24.4%, 57.8%, 73.3% 33.3%, 53.3%, 64.4% 34.1%, 63.6%, 77.3% 0%, 2.2% 26.7%, 51.1% 37.8%, 44.4% 38.6%, 56.8% 13.30% 80.00% 73.30% 81.80%	– – – – – – – – – – – –
Winnette 2022	NCT02974868	PF-06700841	Alopecia Areata	Change in AASIS scores at week 24 Correlation between SALT scores and AASIS scores at baseline Correlation between SALT scores and AASIS scores at week 24	PBO 60 mg QD for 4 ws 30 mg QD for 20 ws PBO 60 mg QD for 4 ws 30 mg QD for 20 ws PBO 60 mg QD for 4 ws 30 mg QD for 20 ws	47 47 47 47 47 47	LS mean difference Pearson correlation Pearson correlation	Ref −1.5 (−2.1, −1.0) Ref 0.18 (0.0119, 0.3325) Ref 0.51 (0.3602, 0.6327)	Ref <0.0001 Ref 0.0359 Ref <0.0001
Unpublished1	NCT03895372	PF-06826647	Plaque psoriasis	Percentage of participants with a PASI 90 response up to week 16 (investigation period)	PBO 50 mg QD 100 mg QD 200 mg QD 400 mg QD	42 22 21 45 41	Risk difference	Ref 8.87 (−4.50, 26.26) 4.76 (−7.07, 21.48) 33.02 (18.01, 47.11) 46.46 (30.62, 60.56)	Ref 0.2621 0.2621 0.0004 <0.0001
Unpublished2	NCT03903822	PF-06700841	Atopic Dermatitis	Percent change from baseline in Eczema Area and Severity Index total score at week 6	PBO QD 0.1% cream QD 0.3% cream QD 1.0% cream QD 3.0% cream QD PBO BID 0.3% cream BID 1.0% cream BID	37 37 36 37 36 36 36 37	LS mean difference LS mean difference	Ref −13.9 (−32.1, 4.3) −20.2 (−38.3, −2.1) −25.6 (−43.3, −8.0) −23.5 (−41.5, −5.5) Ref −11 (−24.3, 2.4) −27.4 (−40.7, −14.1)	Ref 0.104 0.0334 0.0086 0.0158 Ref 0.0879 0.0004
Unpublished3	NCT03715829	PF-06700841	Active Non-segmental Vitiligo	Percent change from baseline in Central Read Facial-Vitiligo Area Scoring Index at week 24	PBO 200 mg + 50 mg QD 100 mg + 50 mg QD 50 mg QD 30 mg QD 10 mg QD	66 65 67 67 50 49	LS mean difference	Ref −23.2 (−32.53, −13.96) −23.2 (−32.53, −13.93) −20.6 (−30.23, −10.93) −16.7 (−27.77, −5.61) −5.1 (−15.02, 4.91)	Ref <0.0001 <0.0001 0.0003 0.0068 0.2015

PASI, Psoriasis Area and Severity Index; sPGA, Static Physician's Global Assessment; SALT, Severity of Alopecia Tool; ACR-20, American College of Rheumatology-20; HAQ-DI, HAQ-Disability Index; SF-36 PCS, Short Form-36 Health Survey Physical Component Summary; DLQI, Dermatology Life Quality Index; BSA, body surface area; AASIS, Alopecia Areata Symptom Impact Scale; PBO, placebo; QD, once daily; BID, twice daily; QOD, every other day; LS mean difference, least-squares mean difference; adjusted OR, adjusted odds ratio; ∗, 90% confidence interval; Ref, reference.

## Discussion

We comprehensively explored the genetic, phenotypic, and clinical data to investigate the efficacy and safety of TYK2 inhibitors. We found consistent evidence supporting the efficacy of TYK2 inhibitors for psoriasis and its related disorders. MR associations of the *TYK2* loss-of-function variant with hypothyroidism, inflammatory bowel disease, primary biliary cirrhosis, and type 1 diabetes supported further investigation of TYK2 inhibitors as a potential treatment for these diseases in future clinical trials. Although only a few clinical trials supported that TYK2 inhibitors appeared to improve disease activity among patients with ulcerative colitis, alopecia areata, atopic dermatitis, or active non-segmental vitiligo, these findings need to be confirmed in larger studies, especially for ulcerative colitis, for which there was conflicting evidence in previous trials. Several potential adverse effects of TYK2 inhibitors, including headache, upper respiratory tract infection, nausea, diarrheal, increased circulating levels of creatinine and liver enzymes, and risk of certain malignant neoplasms, such prostate and breast cancer, should be further explored.

Human genetic data can be used to facilitate drug development and have been found to be effective in many scenarios.^37^ In genome-wide association analyses of common autoimmune diseases, like rheumatoid arthritis,^20^ psoriasis,^19^ multiple sclerosis,^38^ and inflammatory bowel disease,^39^ the *TYK2* gene region has been highlighted, with the allele associated with decreased TYK2 activity showing inverse associations with risk of these diseases. A phenome-wide study on 19 candidate disease targets also indicated that *TYK2* loss-of-function mutation might be associated with several autoimmune diseases,^11^ supporting therapeutic benefit of pharmacological inhibition. Our MR-PheWAS analysis confirmed the inverse associations between genetically proxied TYK2 inhibition and various autoimmune diseases. However, the tissue specific gene expression analysis only validated the inverse effects of genetically proxied TYK2 inhibition on hypothyroidism and psoriasis and its related disorders. In addition, colocalization analysis strengthened the associations for systemic lupus erythematosus, psoriasis, inflammatory bowel disease, and rheumatoid arthritis in appropriate tissues. The findings for psoriasis were supported by RCTs.^1^^,^^2^^,^^40^, ^41^, ^42^, ^43^ The finding for hypothyroidism is in line with a recent MR analysis^11^ and the present analysis went further to support mechanistic relevance specifically in thyroid tissue. For other outcomes associated with genetically proxied TYK2 inhibition, few trials were completed. Thus, the repurposing potential of TYK2 inhibitors for systemic lupus erythematosus and rheumatoid arthritis identified by genetic evidence in our current study needs clinical validation in an RCT setting. Of note, even though MR analysis used a genetic variant to mimic the biological effects of TYK2 inhibitors, several aspects deserve attention when comparing results from the current genetic study and previous trials. First, MR analysis estimated the lifetime exposure to TYK2 inhibitors. Thus, the effect estimates in the current study might be different to that observed in trials that usually last for a short period. In addition, we used loss of function of *TYK2* variant to mimic TYK2 inhibitors without a clear definition of dosage in each arm, which prevented the investigation of the dose–response relationship. Compared to clinical trials, participants of the MR study were more heterogenous, and our MR design is unable to study disease progression. But MR study can usually overcome low treatment adherence (especially when the intervention has serious side-effects) and do not study off-target effects.

TYK2 plays an important role in mediating cytokine signalling and regulating group 1 and 2 cytokine pathways.^44^ Patients carrying *TYK2* loss-of-function mutations are usually characterized by immunodeficiency,^45^ which may increase the risk of health outcomes such as cancer.^46^ From the family of TYK2 inhibitors, JAK inhibitors have been associated with increased risk of certain cancers.^8^^,^^47^ However, whether TYK2 inhibitors increases cancer risk has not been extensively evaluated given lack of long-term RCTs.^2^^,^^48^ Our analysis found inconsistent evidence on the associations of genetically proxied TYK2 inhibition on malignant neoplasms of the prostate or breast. The observed association for prostate cancer is in agreement with a recent MR study where TYK2 inhibition mimicked by a loss-of-function variant in *TYK2* (rs34536443) showed associations with lung cancer, non-Hodgkin lymphoma, and advanced prostate cancer.^9^ Although we observed nominal associations of genetically proxied TYK2 inhibition with prostate and breast cancer risk in both UK Biobank and FinnGen, the tissue-specific gene expression and colocalization analyses did not confirm these associations. From the current evidence, whether TYK2 inhibitor increases the risk of cancer remains undetermined and needs further study, especially in RCTs with a long-term follow-up period.

Other adverse effects reported in previous RCTs include headache, upper respiratory tract infection, nausea, diarrheal, and increased circulating levels of creatinine and liver enzymes.^1^^,^^40^^,^^41^^,^^49^ However, our MR analysis found a contradictory association of genetically proxied TYK2 inhibition with reduced levels of alkaline phosphatase. One *in vivo* study found that deletion of *TYK2* in myeloid cells reduced lipopolysaccharide-induced interleukin 18 production,^50^ which is in line with our MR findings on interleukin 18. In addition, the effects of the *TYK2* loss-of-function variant on sex hormone binding globulin^15^ and insulin-like growth factor-I,^51^^,^^52^ which exerts effects on a wide range of diseases, may also hint at other possible pleiotropic effects related to TYK2 inhibitor use.

The present study has several strengths. Firstly, we explored associations of the *TYK2* loss-of-function mutation with a wide range of disease outcomes in a large biobank and validated the associations in independent populations. Secondly, we used several analytical approaches to examine the associations, and the consistency of results increase confidence in our findings. Thirdly, we conducted a review of RCTs on TYK2 inhibitors to triangulate the evidence. The consistency between findings of the genetic analysis and RCTs further supports the robustness of our conclusions. Limitations also need to be considered when interpreting our findings. Our analysis may have inadequate power for rare diseases and outcomes with low prevalence. For the analyses of biomarkers, we could not compare the results for biomarkers measured in different units across studies with varying sample sizes. Body mass index was adjusted for in the genome-wide association analysis of cytokines and glycaemic traits, which might introduce collider bias in these MR analyses. Although *TYK2* is a protein coding gene, previous studies identified no *cis* signal in this gene affecting gene expression at the genome-wide significance level,^53^ which confined colocalization analysis based on protein quantitative levels. The mediation effect should be interpreted with caution given the strong assumptions to be held under the mediation analysis. In addition, our analysis was majorly based on the European population. Whether our findings can be generalized to other populations needs to be examined in future studies. There was no risk of bias assessment of included trials in the review of TYK2 inhibitors due to limited information on several studies. Thus, whether the summarized evidence from published trials is robust needs to be verified.

In summary, using multiple analytic approaches this study found that genetically proxied TYK2 inhibition was associated with lower risk of psoriasis and its related disorders. The association is largely supported by RCT evidence. The observed associations of TYK2 with other autoimmune diseases, including hypothyroidism, systemic lupus erythematosus and rheumatoid arthritis, should help inform future clinical study design. Finally, potential adverse effects of TYK2 inhibitors, including elevated risk of prostate and breast cancer, should be evaluated in studies with long follow-up duration.

## Contributors

X.L. and S.Y. had full access to all the data in the study and take responsibility for the integrity of the data and the accuracy of the data analysis. X.L., S.L., S.C.L., and E.T. conceived and designed the study. X.L., S.Y., and L.W. undertook the statistical analyses. S.Y. wrote the first draft of the manuscript. X.L. is the study guarantor. S.Y., L.W., H.Z., F.X., X.Z., L.Y., J.S., J.C., H.Y., X.X., Y.Y., A.S., X.S., J.W., D.G., E.T., S.C.L., and X.L. interpreted data, reviewed the paper, and made critical revision of the manuscript for important intellectual content. All authors read and approved the final version of the manuscript.

## Data sharing statement

Data used in this study can be obtained by a reasonable request to corresponding author. This work has been conducted using the UK Biobank Resource. The UK Biobank is an open access resource and bona fide researchers can apply to use the UK Biobank dataset by registering and applying at http://ukbiobank.ac.uk/register-apply/.

## Declaration of interests

DG is employed part-time by Novo Nordisk. The other authors declare no competing interest.
