# Supplementary material for: Mendelian randomization and clinical trial evidence supports TYK2 inhibition as a therapeutic target for autoimmune diseases
Source: eBioMedicine. 2023 Feb 24;89:104488. doi: 10.1016/j.ebiom.2023.104488 (PMC9988426; doi:10.1016/j.ebiom.2023.104488)
Supplement: Supplementary Figs. S1 and S2 and Tables S1–S15 [file mmc1.docx]

Supporting information for

**Mendelian randomization and clinical trial evidence supports TYK2 inhibition as a therapeutic target for autoimmune diseases**

Shuai Yuan, Lijuan Wang, Han Zhang, Fengzhe Xu, Xuan Zhou, Lili Yu, Jing Sun, Jie Chen, Haochao Ying, Xiaolin Xu, Yongfu Yu, Athina Spiliopoulou, Xia Shen, Jim Wilson, Dipender Gill, Evropi Theodoratou, Susanna C. Larsson, Xue Li

**Supplementary Table 1**. Data source for studied biomarkers.

**Supplementary Table 2**. Search strategies.

**Supplementary Table 3**. Characteristics of participants in the UK Biobank.

**Supplementary Table 4.** Outcomes included in the UK Biobank analyses.

**Supplementary Table 5.** Outcomes nominally associated with *TYK2* loss-of-function mutations in MR-PheWAS analysis in UK Biobank.

**Supplementary Table 6.** Mappings of ICD codes to the health outcomes identified by PheWAS analyses.

**Supplementary Table 7.** Results of replication analysis in FinnGen.

**Supplementary Table 8.** Tissue-specific *TYK2* gene expression in relation to risk of diseases.

**Supplementary Table 9.** Cancers associated with *TYK2 gene* in UKB and FinnGen.

**Supplementary Table 10**. *TYK2* gene expression across tissues and F statistics

**Supplementary Table 11**. Associations of additional increase in minor (C) allele of rs34536443 with levels of 231 biomarkers.

**Supplementary Table 12.** Results of SuSiE Colocalization analysis

**Supplementary Table 13.** Mediation analysis for selected biomarkers in the UK Biobank.

**Supplementary Table 14.** Characteristics of 21 included randomized controlled trails in systematic review.

**Supplementary Table 15.** Information on 69 randomized controlled trails registered in *clinicaltrail.gov* database.

**Supplementary Figure 1.** Rs34536443 in *TYK2* gene region differently expressed across 45 tissues.

**Supplementary Figure 2.** Flow chart of study selection for systematic review on trials on TYK2 inhibitor.

**Supplementary Table 1.** Data source for studied biomarkers

| **Biomarker category** | **Source** | **N phenotypes** | **N individuals** | **Population (Ancestry)** | **Adjustments** |
| --- | --- | --- | --- | --- | --- |
| Hematological traits | UK Biobank & INTERVAL [1] | 36 | 173,480 | European | Age, sex |
| Cytokines/Growth factors | YES/FINRISK Studies[2] | 41 | 8,293 | Finnish | Age, sex, BMI |
| Glycemic traits* | MAGIC [3] | 5 | up to 133,010 | European | Age, sex, BMI* |
| Hemodynamic traits | UK Biobank | 3 | 408,228 | White British | Age, sex |
| Serum lipids/metabolites | NMR GWAS[4] | 122 | 24,925 | European | Age, sex |
| Serum/urine biochemistry | UK Biobank | 25 | 353,579 | White British | Age, sex |

* except for HbA1c: the analysis for HbA1c was performed in UK Biobank and we did not adjust for BMI.

**Supplementary Table 2**. Search strategies

| **Database** | **Search strategies** |
| --- | --- |
| **Medline** | 1. "randomized controlled trial".pt. 2. (random$ or placebo$ or single blind$ or double blind$ or triple blind$).ti,ab. 3. (retraction of publication or retracted publication).pt. 4. or/1-3 5. (animals not humans).sh. 6. ((comment or editorial or meta-analysis or practice-guideline or review or letter) not "randomized controlled trial").pt. 7. (random sampl$ or random digit$ or random effect$ or random survey or random regression).ti,ab. not "randomized controlled trial".pt. 8. 4 not (5 or 6 or 7) 9. (TYK2 Kinase or Kinase, TYK2 or Non-Receptor Tyrosine-Protein Kinase TYK2 or Non Receptor Tyrosine Protein Kinase TYK2 or Tyrosine Kinase 2 or protein TYK2 or TYK2 protein).mp. 10. "2-chloro-4-cyano-6-fluoro-N-(2-(2-fluorocyclopropanecarboxamido)pyridin-4-yl)benzamide".mp. 11. ("ropsacitinib" or "3-(cyanomethyl)-3-(4-(6-(1-methylpyrazol-4-yl)pyrazolo(1,5-a)pyrazin-4-yl)pyrazol-1-yl)cyclobutane-1-carbonitrile" or "PF-06826647").mp. 12. ("izencitinib" or "3-((1R,5S)-3-((7-((5-methyl-1H-pyrazol-3-yl)amino)-1,6-naphthyridin-5-yl)amino)-8-azabicyclo(3.2.1)octan-8-yl)propanenitrile" or "3-[(1R,3S,5S)-3-({7-[(5-methyl-1H-pyrazol-3-yl)amino]-1,6-naphthyridin-5-yl}amino)-8-azabicyclo[3.2.1]octan-8-yl]propanenitrile" or "8-azabicyclo(3.2.1)octane-8-propanenitrile, 3-((7-((5-methyl-1h-pyrazol-3-yl)amino)-1,6-naphthyridin-5-yl)amino)-, (3-exo)- " or "TD-1473" or "TD1473").mp. 13. ("Brepocitinib" or "PF-06700841" or "PF-841" or "methanone, ((1S)-2,2-difluorocyclopropyl)(3-(2-((1-methyl-1H-pyrazol-4-yl)amino)-4-pyrimidinyl)-3,8-diazabicyclo(3.2.1)oct-8-yl)- " or "4-[(1R,5S)-8-[(1S)-2,2-difluorocyclopropanecarbonyl]-3,8-diazabicyclo[3.2.1]octan-3-yl]-N-(1-methyl-1H-pyrazol-4-yl)pyrimidin-2-amine").mp. 14. ("deucravacitinib" or "BMS-986165" or "6-cyclopropaneamido-4-{[2-methoxy-3-(1-methyl-1H-1,2,4-triazol-3-yl)phenyl]amino}-N-(2H3)methylpyridazine-3-carboxamide").mp. 15. "BMS-986322".mp. 16. "OST-122".mp. 17. #8 AND (#9 OR #10 OR #11 OR #12 OR #13 OR #14 OR #15 OR #16) |
| **Embase** | 1. (random$ or placebo$ or single blind$ or double blind$ or triple blind$).ti,ab. 2. RETRACTED ARTICLE/ 3. or/1-2 4. (animal$ not human$).sh,hw. 5. (book or conference paper or editorial or letter or review).pt. not exp randomized controlled trial/ 6. (random sampl$ or random digit$ or random effect$ or random survey or random regression). ti,ab. not exp randomized controlled trial/ 7. 3 not (4 or 5 or 6) 8. (TYK2 Kinase or Kinase, TYK2 or Non-Receptor Tyrosine-Protein Kinase TYK2 or Non Receptor Tyrosine Protein Kinase TYK2 or Tyrosine Kinase 2 or protein TYK2 or TYK2 protein).mp. 9. "2-chloro-4-cyano-6-fluoro-N-(2-(2-fluorocyclopropanecarboxamido)pyridin-4-yl)benzamide".mp. 10. ("ropsacitinib" or "3-(cyanomethyl)-3-(4-(6-(1-methylpyrazol-4-yl)pyrazolo(1,5-a)pyrazin-4-yl)pyrazol-1-yl)cyclobutane-1-carbonitrile" or "PF-06826647").mp. 11. ("izencitinib" or "3-((1R,5S)-3-((7-((5-methyl-1H-pyrazol-3-yl)amino)-1,6-naphthyridin-5-yl)amino)-8-azabicyclo(3.2.1)octan-8-yl)propanenitrile" or "3-[(1R,3S,5S)-3-({7-[(5-methyl-1H-pyrazol-3-yl)amino]-1,6-naphthyridin-5-yl}amino)-8-azabicyclo[3.2.1]octan-8-yl]propanenitrile" or "8-azabicyclo(3.2.1)octane-8-propanenitrile, 3-((7-((5-methyl-1h-pyrazol-3-yl)amino)-1,6-naphthyridin-5-yl)amino)-, (3-exo)- " or "TD-1473" or "TD1473").mp. 12. ("Brepocitinib" or "PF-06700841" or "PF-841" or "methanone, ((1S)-2,2-difluorocyclopropyl)(3-(2-((1-methyl-1H-pyrazol-4-yl)amino)-4-pyrimidinyl)-3,8-diazabicyclo(3.2.1)oct-8-yl)- " or "4-[(1R,5S)-8-[(1S)-2,2-difluorocyclopropanecarbonyl]-3,8-diazabicyclo[3.2.1]octan-3-yl]-N-(1-methyl-1H-pyrazol-4-yl)pyrimidin-2-amine").mp. 13. ("deucravacitinib" or "BMS-986165" or "6-cyclopropaneamido-4-{[2-methoxy-3-(1-methyl-1H-1,2,4-triazol-3-yl)phenyl]amino}-N-(2H3)methylpyridazine-3-carboxamide").mp. 14. "BMS-986322".mp. 15. "OST-122".mp. 16. 8 or 9 or 10 or 11 or 12 or 13 or 14 or 15 17. 7 and 16 |
| **ClinicalTrials.gov** | 1. OST-122 2. BMS-986322 3. BMS-986165 4.PF-06700841 5.PF-06826647 6.TD-1473 |

**Supplementary Table 3**. Characteristics of participants in the UK Biobank

| **Characteristics** | **Mean (SD)/ N (%)** |
| --- | --- |
| N | 339,197 |
| Age, mean (SD) | 56.9 (8.0) |
| Female, N (%) | 182,072 (53.7) |
| BMI, kg/m^2^, mean (SD) | 27.4 (4.8) |
| SBP mmHg, mean (SD) | 139.0 (18.7) |
| On antihypertensive medications, N (%) | 74,507 (22.0) |
| LDL cholesterol, mmol/L, mean (SD) | 3.57 (0.9) |
| On lipid-lowering medications, N (%) | 63,652 (18.8) |
| HbA1c, mmol/mol, mean (SD) | 35.95 (6.5) |
| History of diabetes mellitus, N (%) | 26,100 (7.7) |
| Current smoker, N (%) | 34,024 (10.0) |

**Supplementary Table 4.** Outcomes included in the UK Biobank analyses

| **Diagnostic category** | **N Phenotypes** | | | **N cases** | | |
| --- | --- | --- | --- | --- | --- | --- |
|  | **Total** | **Excluded due to power** | **Finally included** | **Median** | **Minimum** | **Maximum** |
| Circulatory system | 145 | 24 | 121 | 1884 | 200 | 96779 |
| Congenital anomalies | 52 | 25 | 27 | 528 | 216 | 1634 |
| Dermatologic | 79 | 28 | 51 | 1042 | 202 | 8884 |
| Digestive | 144 | 23 | 121 | 2153 | 219 | 46601 |
| Endocrine/ Metabolic | 109 | 30 | 79 | 4879 | 203 | 47650 |
| Genitourinary | 150 | 29 | 121 | 1509 | 200 | 21598 |
| Hematopoietic | 50 | 17 | 33 | 798 | 203 | 17972 |
| Infectious disease | 52 | 24 | 28 | 1251 | 245 | 12313 |
| Injuries/poisonings | 77 | 25 | 52 | 1006 | 203 | 25610 |
| Mental disorders | 62 | 21 | 41 | 1126 | 208 | 19366 |
| Musculoskeletal | 120 | 42 | 78 | 1113 | 205 | 55332 |
| Neoplasms | 127 | 20 | 107 | 1538 | 220 | 29647 |
| Neurological | 72 | 23 | 49 | 622 | 209 | 13230 |
| Pregnancy complications | 27 | 13 | 14 | 807 | 216 | 2401 |
| Respiratory | 71 | 14 | 57 | 2047 | 202 | 18440 |
| Sense organs | 105 | 25 | 80 | 943 | 206 | 33716 |
| Symptoms | 31 | 9 | 22 | 1196 | 211 | 21745 |

**Supplementary Table 5.** Outcomes nominally associated with *TYK2* loss-of-function mutations in MR-PheWAS analysis in UK Biobank.

| **Phecode** | **Phenotype** | **Group** | **Cases** | **Controls** | **Beta** | **SE** | ***P*** | **FDR** |
| --- | --- | --- | --- | --- | --- | --- | --- | --- |
| 244.4 | Hypothyroidism NOS | endocrine/metabolic | 18503 | 315717 | -0.176 | 0.028 | 6.23E-10 | TRUE |
| 696.4 | Psoriasis | dermatologic | 2589 | 301676 | -0.458 | 0.084 | 4.11E-08 | TRUE |
| 246 | Other disorders of thyroid | endocrine/metabolic | 21850 | 315717 | -0.141 | 0.026 | 5.26E-08 | TRUE |
| 696.41 | Psoriasis vulgaris | dermatologic | 2751 | 301676 | -0.395 | 0.079 | 5.42E-07 | TRUE |
| 714.1 | Rheumatoid arthritis | musculoskeletal | 5906 | 304719 | -0.236 | 0.050 | 2.79E-06 | TRUE |
| 714 | Rheumatoid arthritis and other inflammatory polyarthropathies | musculoskeletal | 30060 | 304719 | -0.100 | 0.022 | 6.26E-06 | TRUE |
| 696.42 | Psoriatic arthropathy | dermatologic | 929 | 301676 | -0.657 | 0.152 | 1.59E-05 | TRUE |
| 755.1 | Congenital deformities of feet | congenital anomalies | 273 | 336622 | 0.647 | 0.159 | 4.89E-05 | TRUE |
| 70.4 | Chronic hepatitis | infectious diseases | 341 | 330659 | -1.268 | 0.334 | 1.50E-04 | TRUE |
| 555.2 | Ulcerative colitis | digestive | 3269 | 251815 | -0.246 | 0.068 | 2.83E-04 | TRUE |
| 555 | Inflammatory bowel disease and other gastroenteritis and colitis | digestive | 19792 | 251815 | -0.096 | 0.027 | 2.99E-04 | TRUE |
| 557.1 | Celiac disease | digestive | 2185 | 251815 | -0.306 | 0.085 | 3.08E-04 | TRUE |
| 558 | Noninfectious gastroenteritis | digestive | 19875 | 251815 | -0.095 | 0.027 | 3.31E-04 | TRUE |
| 250.1 | Type 1 diabetes | endocrine/metabolic | 2862 | 311499 | -0.260 | 0.073 | 3.68E-04 | TRUE |
| 379 | Other disorders of eye | sense organs | 57586 | 280543 | -0.058 | 0.017 | 4.77E-04 | TRUE |
| 281 | Other deficiency anemia | hematopoietic | 14155 | 310303 | -0.106 | 0.031 | 7.22E-04 | FALSE |
| 571.6 | Primary biliary cirrhosis | digestive | 330 | 322199 | -1.011 | 0.302 | 8.13E-04 | FALSE |
| 356 | Hereditary and idiopathic peripheral neuropathy | neurological | 224 | 333383 | 0.577 | 0.181 | 1.47E-03 | FALSE |
| 585.2 | Renal failure NOS | genitourinary | 21591 | 309969 | -0.082 | 0.026 | 1.58E-03 | FALSE |
| 81 | Infection/inflammation of internal prosthetic device; implant; and graft | infectious diseases | 3432 | 329979 | -0.202 | 0.065 | 1.94E-03 | FALSE |
| 300 | Anxiety, phobic and dissociative disorders | mental disorders | 566 | 269431 | -0.580 | 0.190 | 2.21E-03 | FALSE |
| 250.7 | Diabetic retinopathy | endocrine/metabolic | 2132 | 317402 | -0.255 | 0.085 | 2.55E-03 | FALSE |
| 401.1 | Essential hypertension | circulatory system | 96557 | 241390 | -0.043 | 0.014 | 2.96E-03 | FALSE |
| 401 | Hypertension | circulatory system | 96780 | 241390 | -0.043 | 0.014 | 3.17E-03 | FALSE |
| 458.1 | Orthostatic hypotension | circulatory system | 3385 | 188944 | -0.193 | 0.066 | 3.59E-03 | FALSE |
| 459.9 | Circulatory disease NEC | circulatory system | 148868 | 188944 | -0.038 | 0.013 | 3.69E-03 | FALSE |
| 709.2 | Sicca syndrome | dermatologic | 718 | 206518 | -0.459 | 0.158 | 3.76E-03 | FALSE |
| 697 | Sarcoidosis | dermatologic | 701 | 329167 | -0.455 | 0.160 | 4.35E-03 | FALSE |
| 835 | Internal derangement of knee | injuries & poisonings | 15047 | 320882 | -0.085 | 0.030 | 4.40E-03 | FALSE |
| 747.2 | Congenital anomalies of peripheral vascular system | congenital anomalies | 220 | 333403 | 0.524 | 0.187 | 5.11E-03 | FALSE |
| 274.1 | Gout | endocrine/metabolic | 5936 | 331400 | -0.137 | 0.049 | 5.19E-03 | FALSE |
| 250.23 | Type 2 diabetes with ophthalmic manifestations | endocrine/metabolic | 2274 | 311499 | -0.226 | 0.081 | 5.27E-03 | FALSE |
| 800.3 | Fracture of tibia and fibula | injuries & poisonings | 2367 | 311878 | 0.183 | 0.066 | 5.73E-03 | FALSE |
| 722.6 | Degeneration of intervertebral disc | musculoskeletal | 4147 | 299873 | 0.141 | 0.051 | 5.85E-03 | FALSE |
| 251.1 | Hypoglycemia | endocrine/metabolic | 1861 | 307992 | -0.247 | 0.090 | 6.13E-03 | FALSE |
| 745 | Pain in joint | musculoskeletal | 10435 | 327737 | 0.090 | 0.033 | 6.84E-03 | FALSE |
| 280.1 | Iron deficiency anemias, unspecified or not due to blood loss | hematopoietic | 12467 | 310303 | -0.089 | 0.033 | 6.90E-03 | FALSE |
| 172.2 | Other non-epithelial cancer of skin | neoplasms | 23987 | 309145 | 0.061 | 0.023 | 7.83E-03 | FALSE |
| 585.3 | Chronic renal failure [CKD] | genitourinary | 6092 | 309969 | -0.124 | 0.048 | 9.67E-03 | FALSE |
| 366.2 | Senile cataract | sense organs | 18542 | 301753 | -0.072 | 0.028 | 9.94E-03 | FALSE |
| 555.1 | Regional enteritis | digestive | 1933 | 251815 | -0.224 | 0.087 | 1.00E-02 | FALSE |
| 447.1 | Stricture of artery | circulatory system | 1149 | 324290 | -0.303 | 0.118 | 1.03E-02 | FALSE |
| 496 | Chronic airway obstruction | respiratory | 12605 | 296427 | -0.085 | 0.033 | 1.05E-02 | FALSE |
| 242 | Thyrotoxicosis with or without goiter | endocrine/metabolic | 2012 | 315717 | -0.217 | 0.085 | 1.07E-02 | FALSE |
| 427.61 | Supraventricular premature beats | circulatory system | 231 | 300606 | -0.848 | 0.332 | 1.08E-02 | FALSE |
| 263 | Other nutritional deficiency | endocrine/metabolic | 5157 | 331444 | -0.131 | 0.052 | 1.10E-02 | FALSE |
| 187.2 | Malignant neoplasm of testis | neoplasms | 7357 | 323893 | 0.100 | 0.040 | 1.12E-02 | FALSE |
| 575.6 | Cholesterolosis of gallbladder | digestive | 570 | 316601 | 0.322 | 0.127 | 1.16E-02 | FALSE |
| 371.3 | Inflammation of eyelids | sense organs | 2711 | 326486 | -0.181 | 0.072 | 1.24E-02 | FALSE |
| 782.3 | Edema | symptoms | 3794 | 334097 | -0.151 | 0.061 | 1.27E-02 | FALSE |
| 694.2 | Other dyschromia | dermatologic | 1042 | 329167 | 0.241 | 0.097 | 1.27E-02 | FALSE |
| 198.4 | Secondary malignant neoplasm of liver | neoplasms | 4750 | 217117 | 0.121 | 0.049 | 1.31E-02 | FALSE |
| 379.3 | Aphakia and other disorders of lens | sense organs | 36145 | 280543 | -0.051 | 0.020 | 1.32E-02 | FALSE |
| 613.9 | Breast disorder NOS | genitourinary | 5311 | 332636 | 0.114 | 0.046 | 1.35E-02 | FALSE |
| 187.1 | Malignant neoplasm of unspecified male genital organ | neoplasms | 13386 | 323893 | 0.075 | 0.030 | 1.37E-02 | FALSE |
| 429.2 | Abnormal function study of cardiovascular system | circulatory system | 864 | 326516 | 0.258 | 0.106 | 1.46E-02 | FALSE |
| 165.1 | Cancer of bronchus; lung | neoplasms | 3772 | 333288 | 0.131 | 0.054 | 1.58E-02 | FALSE |
| 716.2 | Unspecified monoarthritis | musculoskeletal | 22081 | 246702 | -0.062 | 0.026 | 1.59E-02 | FALSE |
| 560.2 | Impaction of intestine | digestive | 492 | 251815 | 0.327 | 0.136 | 1.62E-02 | FALSE |
| 281.12 | Other vitamin B12 deficiency anemia | hematopoietic | 259 | 310303 | -0.701 | 0.295 | 1.73E-02 | FALSE |
| 41.1 | Staphylococcus infections | infectious diseases | 4062 | 315572 | -0.136 | 0.058 | 1.91E-02 | FALSE |
| 367.2 | Astigmatism | sense organs | 2263 | 331987 | -0.187 | 0.080 | 1.95E-02 | FALSE |
| 426.21 | First degree AV block | circulatory system | 2455 | 300606 | -0.177 | 0.076 | 1.96E-02 | FALSE |
| 585.1 | Acute renal failure | genitourinary | 13026 | 309969 | -0.075 | 0.033 | 2.07E-02 | FALSE |
| 728 | Disorders of muscle, ligament, and fascia | musculoskeletal | 352 | 293013 | -0.539 | 0.234 | 2.15E-02 | FALSE |
| 278.1 | Obesity | endocrine/metabolic | 21503 | 316519 | -0.064 | 0.028 | 2.19E-02 | FALSE |
| 571.8 | Liver abscess and sequelae of chronic liver disease | digestive | 1062 | 322199 | -0.274 | 0.121 | 2.32E-02 | FALSE |
| 261.2 | Vitamin B-complex deficiencies | endocrine/metabolic | 1964 | 331444 | -0.194 | 0.086 | 2.36E-02 | FALSE |
| 359.2 | Myopathy | neurological | 1880 | 333383 | -0.198 | 0.088 | 2.41E-02 | FALSE |
| 165 | Cancer within the respiratory system | neoplasms | 4859 | 333288 | 0.107 | 0.048 | 2.61E-02 | FALSE |
| 451 | Phlebitis and thrombophlebitis | circulatory system | 562 | 299434 | -0.387 | 0.174 | 2.61E-02 | FALSE |
| 394.3 | Aortic valve disease | circulatory system | 2453 | 325604 | -0.168 | 0.076 | 2.63E-02 | FALSE |
| 427.42 | Cardiac arrest | circulatory system | 1818 | 300606 | -0.196 | 0.089 | 2.69E-02 | FALSE |
| 578.9 | Hemorrhage of gastrointestinal tract | digestive | 9228 | 309470 | -0.084 | 0.038 | 2.71E-02 | FALSE |
| 276.14 | Hypopotassemia | endocrine/metabolic | 4068 | 321139 | -0.128 | 0.058 | 2.76E-02 | FALSE |
| 772 | Symptoms of the muscles | symptoms | 988 | 337145 | -0.275 | 0.125 | 2.78E-02 | FALSE |
| 569 | Other disorders of intestine | digestive | 96364 | 239026 | -0.030 | 0.014 | 2.81E-02 | FALSE |
| 290 | Delirium dementia and amnestic and other cognitive disorders | mental disorders | 1817 | 323003 | -0.195 | 0.089 | 2.82E-02 | FALSE |
| 335 | Multiple sclerosis | neurological | 1301 | 285132 | -0.234 | 0.107 | 2.83E-02 | FALSE |
| 297 | Suicidal ideation or attempt | mental disorders | 275 | 269431 | -0.651 | 0.299 | 2.92E-02 | FALSE |
| 727.1 | Synovitis and tenosynovitis | musculoskeletal | 3388 | 293013 | -0.138 | 0.063 | 2.94E-02 | FALSE |
| 557 | Intestinal malabsorption (non-celiac) | digestive | 456 | 251815 | -0.417 | 0.194 | 3.16E-02 | FALSE |
| 722 | Intervertebral disc disorders | musculoskeletal | 7574 | 299873 | 0.083 | 0.039 | 3.36E-02 | FALSE |
| 185 | Cancer of prostate | neoplasms | 13467 | 308080 | 0.065 | 0.031 | 3.39E-02 | FALSE |
| 522 | Diseases of pulp and periapical tissues | digestive | 610 | 319989 | 0.266 | 0.126 | 3.42E-02 | FALSE |
| 159.3 | Malignant neoplasm of gallbladder and extrahepatic bile ducts | neoplasms | 392 | 314914 | -0.452 | 0.214 | 3.43E-02 | FALSE |
| 689 | Disorder of skin and subcutaneous tissue NOS | dermatologic | 51221 | 286951 | -0.036 | 0.017 | 3.45E-02 | FALSE |
| 277 | Other disorders of metabolism | endocrine/metabolic | 63222 | 274937 | -0.034 | 0.016 | 3.53E-02 | FALSE |
| 614.4 | Inflammatory diseases of uterus, except cervix | genitourinary | 270 | 329253 | 0.376 | 0.179 | 3.58E-02 | FALSE |
| 722.9 | Other and unspecified disc disorder | musculoskeletal | 6226 | 299873 | 0.089 | 0.043 | 3.73E-02 | FALSE |
| 916 | Contusion | injuries & poisonings | 2496 | 335676 | -0.155 | 0.075 | 3.83E-02 | FALSE |
| 427.41 | Ventricular fibrillation and flutter | circulatory system | 568 | 300606 | -0.351 | 0.170 | 3.86E-02 | FALSE |
| 752 | Nervous system congenital anomalies | congenital anomalies | 316 | 337503 | 0.346 | 0.168 | 3.98E-02 | FALSE |
| 174.1 | Breast cancer [female] | neoplasms | 4133 | 306631 | 0.107 | 0.052 | 4.03E-02 | FALSE |
| 195.1 | Malignant neoplasm, other | neoplasms | 115707 | 217117 | 0.026 | 0.013 | 4.06E-02 | FALSE |
| 198.3 | Secondary malignant neoplasm of digestive systems | neoplasms | 2804 | 217117 | 0.128 | 0.063 | 4.13E-02 | FALSE |
| 396 | Abnormal heart sounds | circulatory system | 1927 | 325604 | 0.150 | 0.074 | 4.25E-02 | FALSE |
| 519 | Other diseases of respiratory system, not elsewhere classified | respiratory | 78158 | 259141 | -0.029 | 0.014 | 4.25E-02 | FALSE |
| 287.3 | Thrombocytopenia | hematopoietic | 2266 | 334085 | -0.157 | 0.078 | 4.45E-02 | FALSE |
| 495 | Asthma | respiratory | 30136 | 296427 | -0.043 | 0.021 | 4.48E-02 | FALSE |
| 341 | Other demyelinating diseases of central nervous system | neurological | 1512 | 285132 | -0.195 | 0.097 | 4.53E-02 | FALSE |
| 709.7 | Unspecified diffuse connective tissue disease | dermatologic | 128637 | 206518 | -0.026 | 0.013 | 4.54E-02 | FALSE |
| 756.5 | Congenital osteodystrophies | congenital anomalies | 596 | 336622 | -0.327 | 0.164 | 4.57E-02 | FALSE |
| 386 | Vertiginous syndromes and other disorders of vestibular system | sense organs | 3077 | 328429 | -0.132 | 0.066 | 4.76E-02 | FALSE |
| 284 | Aplastic anemia | hematopoietic | 18390 | 310303 | -0.053 | 0.027 | 4.91E-02 | FALSE |

FDR, false discovery rate; OR, odds ratio; SE, standard error.

**Supplementary Table 6.** Mappings of ICD codes to the health outcomes identified by PheWAS analyses.

| **Phecode** | **Phenotype** | **ICD-10** | **ICD-9** |
| --- | --- | --- | --- |
| 696.4 | Psoriasis | L40-L40.9, M07.0, M07.2, M07.3 | 696, 696.1, 696.8 |
| 244 | Hypothyroidism | E00-E00.2, E00.9, E01.8, E02, E03.0-E03.3, E03.8, E03.9, E89.0 | 243, 244.1-244.4 |
| 714.1 | Rheumatoid arthritis | J99.0, M05.0, M05.1, M05.3, M05.9-M06.3, M06.8, M06.9 | 714, 714.1, 714.2, 714.81 |
| 696.41 | Psoriasis vulgaris | L40.0-L40.4, L40.8, L40.9 | 696.1 |
| 555 | Inflammatory bowel disease and other gastroenteritis and colitis | K50-K50.1, K50.8, K50.9, K51-K51.5, K51.8, K51.9 | 555, 555.1, 555.2, 555.9, 556-556.6, 556.8, 556.9 |
| 755.1 | Congenital deformities of feet | Q66-Q66.9, Q72.7 | 754.5, 754.51-754.53, 754.59, 754.6, 754.61, 754.62, 754.69, 754.7, 754.71, 754.79, 755.67 |
| 555.2 | Ulcerative colitis | K51-K51.9 | 556-556.6, 556.8, 556.9 |
| 70.4 | Chronic hepatitis | K71.5, K73-K73.2, K73.8, K73.9, K75.4 | 571.4, 571.41, 571.42, 571.49 |
| 709 | Diffuse diseases of connective tissue | L94.3, M33-M33.2, M33.9-M34.2, M34.8-M35.1, M65.3, N16.4 | 710, 710.1-710.4, 710.8, 710.9, 517.2 |
| 592.11 | Acute cystitis | N30.0 | 595 |
| 571.6 | Primary biliary cirrhosis | K74.3-K74.5 | 571.6 |
| 585.3 | Chronic renal failure [CKD] | N18, N18.0, N18.8, N18.9, Y60.2, Y61.2, Y62.0, Y84.1, Z49.1, Z49.2, Z99.2 | 585, 585.0, 585.3-585.6, 585.9, 792.5, 996.73 |
| 696.42 | Psoriatic arthropathy | L40.5, M07.0, M07.2, M07.3 | 696 |
| 250.1 | Type 1 diabetes | E10-E10.4, E10.6-E10.9 | 250.01-250.83 |

**Supplementary Table 7.** Results of replication analysis in FinnGen

| **Outcome in UK Biobank** | **Outcome in FinnGen** | **Cases** | **Controls** | **Beta** | **SE** | ***P* value** |
| --- | --- | --- | --- | --- | --- | --- |
| Hypothyroidism | Hypothyroidism | 32925 | 65456 | -0.185 | 0.037 | 7.26E-07 |
| Psoriasis | Psoriasis | 5621 | 252323 | -0.241 | 0.060 | 5.07E-05 |
| Rheumatoid arthritis | Rheumatoid arthritis | 7909 | 173414 | -0.311 | 0.053 | 3.31E-09 |
| Psoriasis vulgaris | Psoriasis vulgaris | 3508 | 252323 | -0.281 | 0.074 | 1.53E-04 |
| Inflammatory bowel disease | Inflammatory bowel disease | 7206 | 253199 | -0.170 | 0.052 | 1.19E-03 |
| Psoriatic arthropathy | Psoriatic arthropathy | 1935 | 173414 | -0.447 | 0.101 | 8.87E-06 |
| Congenital deformities of feet | Congenital deformities of feet | 415 | 258787 | 0.097 | 0.206 | 6.37E-01 |
| Ulcerative colitis | Ulcerative colitis | 5349 | 249705 | -0.181 | 0.060 | 2.52E-03 |
| Chronic hepatitis | Chronic hepatitis NAS | 233 | 260172 | -0.665 | 0.273 | 1.49E-02 |
| Type 1 diabetes | Type 1 diabetes | 7609 | 215160 | -0.257 | 0.058 | 9.27E-06 |
| Diffuse diseases of connective tissue | Systemic connective tissue disorders | 7055 | 253350 | -0.149 | 0.051 | 3.80E-03 |
| Acute cystitis | Cystitis | 9918 | 231480 | -0.081 | 0.043 | 6.36E-02 |
| Primary biliary cirrhosis | Primary biliary chirrosis | 346 | 207748 | -0.781 | 0.225 | 5.07E-04 |
| Chronic kidney disease | Chronic kidney disease | 4959 | 252950 | -0.025 | 0.061 | 6.88E-01 |
| Congenital anomalies of stomach | NA | NA | NA | NA | NA | NA |
| Celiac disease | NA | NA | NA | NA | NA | NA |

NA, not available; SE, standard error.

**Supplementary Table 8.** Tissue-specific *TYK2* gene expression in relation to risk of diseases

| **Phecode** | **Description** | **Tissue** | **Group** | **Cases** | **Participants** | **Beta** | **SE** | **OR** | **P** |
| --- | --- | --- | --- | --- | --- | --- | --- | --- | --- |
| 244 | Hypothyroidism | BFCB | endocrine/metabolic | 19428 | 335158 | -0.49 | 0.11 | 0.61 | 1.25E-05 |
| 244 | Hypothyroidism | BNABG | endocrine/metabolic | 19428 | 335158 | -0.23 | 0.08 | 0.80 | 3.99E-03 |
| 244 | Hypothyroidism | Brain_Hippocampus | endocrine/metabolic | 19428 | 335158 | -0.34 | 0.12 | 0.71 | 3.99E-03 |
| 244.4 | Hypothyroidism NOS | BFCB | endocrine/metabolic | 18503 | 334233 | -0.51 | 0.12 | 0.60 | 1.10E-05 |
| 244.4 | Hypothyroidism NOS | BNABG | endocrine/metabolic | 18503 | 334233 | -0.24 | 0.08 | 0.78 | 2.56E-03 |
| 244.4 | Hypothyroidism NOS | Brain_Hippocampus | endocrine/metabolic | 18503 | 334233 | -0.36 | 0.12 | 0.70 | 2.56E-03 |
| 244.4 | Hypothyroidism NOS | CS | endocrine/metabolic | 18503 | 334233 | -0.16 | 0.08 | 0.85 | 4.73E-02 |
| 244.4 | Hypothyroidism NOS | Liver | endocrine/metabolic | 18503 | 334233 | -0.08 | 0.04 | 0.93 | 4.73E-02 |
| 244.4 | Hypothyroidism NOS | Thyroid | endocrine/metabolic | 18503 | 334233 | -0.15 | 0.07 | 0.86 | 4.73E-02 |
| 250.1 | Type 1 diabetes | BFCB | endocrine/metabolic | 2903 | 315164 | -0.61 | 0.28 | 0.54 | 3.06E-02 |
| 250.1 | Type 1 diabetes | BNABG | endocrine/metabolic | 2903 | 315164 | -0.42 | 0.20 | 0.66 | 3.42E-02 |
| 250.1 | Type 1 diabetes | Brain_Hippocampus | endocrine/metabolic | 2903 | 315164 | -0.62 | 0.29 | 0.54 | 3.42E-02 |
| 356 | Hereditary and idiopathic peripheral neuropathy | BSN | neurological | 224 | 335335 | 5.19 | 2.43 | 180.29 | 3.24E-02 |
| 555 | Inflammatory bowel disease and other gastroenteritis and colitis | BFCB | digestive | 4851 | 279185 | -0.67 | 0.22 | 0.51 | 2.17E-03 |
| 555 | Inflammatory bowel disease and other gastroenteritis and colitis | BNABG | digestive | 4851 | 279185 | -0.34 | 0.15 | 0.71 | 2.34E-02 |
| 555 | Inflammatory bowel disease and other gastroenteritis and colitis | Brain_Hippocampus | digestive | 4851 | 279185 | -0.51 | 0.23 | 0.60 | 2.34E-02 |
| 557.1 | Celiac disease | BNABG | digestive | 2185 | 276519 | -0.47 | 0.23 | 0.63 | 3.76E-02 |
| 557.1 | Celiac disease | Brain_Hippocampus | digestive | 2185 | 276519 | -0.70 | 0.34 | 0.50 | 3.76E-02 |
| 585.3 | Chronic renal failure [CKD] | BC | genitourinary | 11321 | 322394 | -0.35 | 0.09 | 0.71 | 1.36E-04 |
| 585.3 | Chronic renal failure [CKD] | BCH | genitourinary | 11321 | 322394 | 0.11 | 0.04 | 1.11 | 3.06E-03 |
| 585.3 | Chronic renal failure [CKD] | Brain_Cerebellum | genitourinary | 11321 | 322394 | 0.09 | 0.03 | 1.09 | 7.60E-03 |
| 585.3 | Chronic renal failure [CKD] | CS | genitourinary | 11321 | 322394 | 0.29 | 0.10 | 1.33 | 4.73E-03 |
| 585.3 | Chronic renal failure [CKD] | Liver | genitourinary | 11321 | 322394 | 0.14 | 0.05 | 1.15 | 4.73E-03 |
| 585.3 | Chronic renal failure [CKD] | Skin | genitourinary | 11321 | 322394 | 0.29 | 0.10 | 1.34 | 4.38E-03 |
| 585.3 | Chronic renal failure [CKD] | Thyroid | genitourinary | 11321 | 322394 | 0.26 | 0.09 | 1.30 | 4.73E-03 |
| 696 | Psoriasis and related disorders | BFCB | dermatologic | 3500 | 324850 | -1.30 | 0.26 | 0.27 | 8.17E-07 |
| 696 | Psoriasis and related disorders | BNABG | dermatologic | 3500 | 324850 | -0.53 | 0.18 | 0.59 | 3.02E-03 |
| 696 | Psoriasis and related disorders | Brain_Cerebellum | dermatologic | 3500 | 324850 | -0.17 | 0.06 | 0.84 | 3.63E-03 |
| 696 | Psoriasis and related disorders | Brain_Hippocampus | dermatologic | 3500 | 324850 | -0.79 | 0.27 | 0.45 | 3.02E-03 |
| 696 | Psoriasis and related disorders | CS | dermatologic | 3500 | 324850 | -0.74 | 0.17 | 0.48 | 2.57E-05 |
| 696 | Psoriasis and related disorders | Liver | dermatologic | 3500 | 324850 | -0.36 | 0.08 | 0.70 | 2.57E-05 |
| 696 | Psoriasis and related disorders | Skin | dermatologic | 3500 | 324850 | -0.72 | 0.18 | 0.49 | 4.50E-05 |
| 696 | Psoriasis and related disorders | Thyroid | dermatologic | 3500 | 324850 | -0.68 | 0.16 | 0.50 | 2.57E-05 |
| 696.4 | Psoriasis | BCH | dermatologic | 3416 | 324766 | -0.13 | 0.06 | 0.88 | 4.05E-02 |
| 696.4 | Psoriasis | BFCB | dermatologic | 3416 | 324766 | -1.30 | 0.27 | 0.27 | 1.09E-06 |
| 696.4 | Psoriasis | BNABG | dermatologic | 3416 | 324766 | -0.54 | 0.18 | 0.58 | 2.69E-03 |
| 696.4 | Psoriasis | Brain_Cerebellum | dermatologic | 3416 | 324766 | -0.18 | 0.06 | 0.83 | 1.97E-03 |
| 696.4 | Psoriasis | Brain_Hippocampus | dermatologic | 3416 | 324766 | -0.81 | 0.27 | 0.44 | 2.69E-03 |
| 696.4 | Psoriasis | CS | dermatologic | 3416 | 324766 | -0.76 | 0.18 | 0.47 | 1.57E-05 |
| 696.4 | Psoriasis | Liver | dermatologic | 3416 | 324766 | -0.37 | 0.09 | 0.69 | 1.57E-05 |
| 696.4 | Psoriasis | Skin | dermatologic | 3416 | 324766 | -0.75 | 0.18 | 0.47 | 2.65E-05 |
| 696.4 | Psoriasis | Thyroid | dermatologic | 3416 | 324766 | -0.71 | 0.16 | 0.49 | 1.57E-05 |
| 696.41 | Psoriasis vulgaris | BFCB | dermatologic | 2734 | 324084 | -1.23 | 0.30 | 0.29 | 3.53E-05 |
| 696.41 | Psoriasis vulgaris | BNABG | dermatologic | 2734 | 324084 | -0.50 | 0.20 | 0.61 | 1.36E-02 |
| 696.41 | Psoriasis vulgaris | Brain_Cerebellum | dermatologic | 2734 | 324084 | -0.13 | 0.07 | 0.88 | 4.21E-02 |
| 696.41 | Psoriasis vulgaris | Brain_Hippocampus | dermatologic | 2734 | 324084 | -0.75 | 0.30 | 0.47 | 1.36E-02 |
| 696.41 | Psoriasis vulgaris | CS | dermatologic | 2734 | 324084 | -0.66 | 0.20 | 0.52 | 8.73E-04 |
| 696.41 | Psoriasis vulgaris | Liver | dermatologic | 2734 | 324084 | -0.32 | 0.10 | 0.73 | 8.73E-04 |
| 696.41 | Psoriasis vulgaris | Skin | dermatologic | 2734 | 324084 | -0.64 | 0.20 | 0.53 | 1.37E-03 |
| 696.41 | Psoriasis vulgaris | Thyroid | dermatologic | 2734 | 324084 | -0.61 | 0.18 | 0.54 | 8.73E-04 |
| 696.42 | Psoriatic arthropathy | BC | dermatologic | 924 | 322274 | 0.88 | 0.31 | 2.41 | 4.15E-03 |
| 696.42 | Psoriatic arthropathy | BCH | dermatologic | 924 | 322274 | -0.25 | 0.12 | 0.78 | 4.20E-02 |
| 696.42 | Psoriatic arthropathy | BFCB | dermatologic | 924 | 322274 | -1.71 | 0.52 | 0.18 | 9.20E-04 |
| 696.42 | Psoriatic arthropathy | BNABG | dermatologic | 924 | 322274 | -0.91 | 0.35 | 0.40 | 9.76E-03 |
| 696.42 | Psoriatic arthropathy | Brain_Cerebellum | dermatologic | 924 | 322274 | -0.32 | 0.11 | 0.72 | 4.29E-03 |
| 696.42 | Psoriatic arthropathy | Brain_Hippocampus | dermatologic | 924 | 322274 | -1.35 | 0.52 | 0.26 | 9.76E-03 |
| 696.42 | Psoriatic arthropathy | CS | dermatologic | 924 | 322274 | -1.05 | 0.34 | 0.35 | 1.96E-03 |
| 696.42 | Psoriatic arthropathy | Liver | dermatologic | 924 | 322274 | -0.51 | 0.16 | 0.60 | 1.96E-03 |
| 696.42 | Psoriatic arthropathy | Skin | dermatologic | 924 | 322274 | -1.05 | 0.34 | 0.35 | 2.08E-03 |
| 696.42 | Psoriatic arthropathy | Thyroid | dermatologic | 924 | 322274 | -0.97 | 0.31 | 0.38 | 1.96E-03 |
| 709 | Diffuse diseases of connective tissue | BSN | dermatologic | 1175 | 325726 | 2.48 | 1.14 | 11.99 | 2.99E-02 |
| 714 | Rheumatoid arthritis and other inflammatory polyarthropathies | CS | musculoskeletal | 4752 | 332339 | -0.32 | 0.15 | 0.73 | 3.57E-02 |
| 714 | Rheumatoid arthritis and other inflammatory polyarthropathies | Liver | musculoskeletal | 4752 | 332339 | -0.15 | 0.07 | 0.86 | 3.57E-02 |
| 714 | Rheumatoid arthritis and other inflammatory polyarthropathies | Skin | musculoskeletal | 4752 | 332339 | -0.30 | 0.15 | 0.74 | 4.54E-02 |
| 714 | Rheumatoid arthritis and other inflammatory polyarthropathies | Thyroid | musculoskeletal | 4752 | 332339 | -0.29 | 0.14 | 0.75 | 3.57E-02 |
| 714.1 | Rheumatoid arthritis | BSN | musculoskeletal | 4211 | 331798 | 1.26 | 0.63 | 3.54 | 4.37E-02 |

OR, odds ratio; SE, standard error.

**Supplementary Table 9.** Cancers associated with *TYK2 gene* in UKB and FinnGen

| **Phecode** | **Phenotype** | **UK Biobank** | | | | | | **FinnGen** | | | | | |
| --- | --- | --- | --- | --- | --- | --- | --- | --- | --- | --- | --- | --- | --- |
|  |  | **Cases** | **Controls** | **Beta** | **SE** | **OR** | ***P*** | **Cases** | **Controls** | **Beta** | **SE** | **OR** | ***P*** |
| 187.2 | Malignant neoplasm of testis | 7357 | 324127 | 0.10 | 0.04 | 1.11 | 0.011 | 268 | 87089 | 0.20 | 0.25 | 1.22 | 0.441 |
| 187 | Cancer of male genital organs | 7518 | 324127 | 0.10 | 0.04 | 1.10 | 0.011 | 9025 | 87089 | 0.13 | 0.05 | 1.14 | 0.013 |
| 165.1 | Cancer of bronchus and lung | 3772 | 333464 | 0.13 | 0.05 | 1.14 | 0.016 | 3061 | 204021 | -0.05 | 0.08 | 0.95 | 0.528 |
| 174 | Breast cancer | 11293 | 310156 | 0.07 | 0.03 | 1.07 | 0.028 | 11573 | 116981 | 0.09 | 0.04 | 1.09 | 0.048 |
| 185 | Cancer of prostate | 13467 | 308326 | 0.07 | 0.03 | 1.07 | 0.034 | 8709 | 87089 | 0.14 | 0.02 | 1.15 | 0.009 |

OR, odds ratio; SE, standard error.

**Supplementary Table 10. *TYK2* gene expression across tissues and F statistics**

| **Tissue** | **N_SNPs** | **Top SNP** | **P** | **R^2^** | **F statistic** |
| --- | --- | --- | --- | --- | --- |
| Adipose_Subcutaneous | 581 | rs280497 | 2.75E-15 | 0.10 | 62.22 |
| Adipose_Visceral_Omentum | 469 | rs280497 | 5.64E-09 | 0.07 | 33.81 |
| Adrenal_Gland | 233 | rs12720349 | 1.47E-12 | 0.18 | 49.65 |
| Artery_Aorta | 387 | rs12720328 | 1.20E-05 | 0.05 | 19.06 |
| Artery_Tibial | 584 | rs11669299 | 1.74E-07 | 0.04 | 27.21 |
| Brain_Cerebellar_Hemisphere | 175 | rs280497 | 8.56E-12 | 0.21 | 46.10 |
| Brain_Cerebellum | 209 | rs280497 | 4.77E-15 | 0.23 | 60.77 |
| Brain_Cortex | 205 | rs4804134 | 4.35E-09 | 0.14 | 34.12 |
| Brain_Hypothalamus | 170 | rs118115488 | 8.86E-06 | 0.10 | 19.51 |
| Breast_Mammary_Tissue | 396 | rs280497 | 1.37E-09 | 0.08 | 36.52 |
| Cells_Cultured_fibroblasts | 483 | rs6511695 | 9.10E-16 | 0.12 | 64.35 |
| Colon_Sigmoid | 318 | rs280497 | 7.97E-10 | 0.11 | 37.53 |
| Esophagus_Gastroesophageal_Junction | 330 | rs55765999 | 7.22E-07 | 0.07 | 24.41 |
| Esophagus_Mucosa | 497 | rs12720299 | 2.84E-07 | 0.05 | 26.25 |
| Esophagus_Muscularis | 465 | rs6511698 | 4.83E-07 | 0.05 | 25.22 |
| Heart_Atrial_Appendage | 372 | rs280497 | 4.80E-05 | 0.04 | 16.44 |
| Liver | 208 | rs280497 | 8.31E-10 | 0.15 | 37.32 |
| Lung | 515 | rs280497 | 2.00E-10 | 0.07 | 40.31 |
| Nerve_Tibial | 532 | rs280497 | 2.83E-16 | 0.11 | 66.67 |
| Pancreas | 305 | rs280497 | 3.95E-09 | 0.10 | 34.42 |
| Pituitary | 237 | rs11880408 | 1.99E-06 | 0.09 | 22.41 |
| Skin_Not_Sun_Exposed_Suprapubic | 517 | rs280497 | 9.90E-14 | 0.10 | 55.17 |
| Skin_Sun_Exposed_Lower_leg | 605 | rs35251378 | 4.42E-11 | 0.07 | 43.27 |
| Small_Intestine_Terminal_Ileum | 174 | rs73508685 | 2.17E-05 | 0.09 | 17.83 |
| Spleen | 227 | rs280497 | 2.21E-10 | 0.15 | 39.92 |
| Stomach | 324 | rs280497 | 3.24E-05 | 0.05 | 17.17 |
| Testis | 322 | rs11878725 | 4.43E-06 | 0.06 | 20.94 |
| Thyroid | 574 | rs280497 | 1.59E-12 | 0.08 | 49.75 |
| Whole_Blood | 670 | rs280497 | 4.80E-16 | 0.09 | 65.68 |

SNP, single nucleotide polymorphism.

**Supplementary Table 11. Results of SuSiE Colocalization analysis**

| **Disease** | **Tissue** | **N_SNPs** | **SNP1** | **SNP2** | **PP.H0.abf** | **PP.H1.abf** | **PP.H2.abf** | **PP.H3.abf** | **PP.H4.abf** |
| --- | --- | --- | --- | --- | --- | --- | --- | --- | --- |
| CD | Skin_Not_Sun_Exposed_Suprapubic | 101 | rs61451658 | rs28378712 | 0.00E+00 | 0.00E+00 | 2.52E-39 | 8.67E-03 | 0.99 |
| UC | Skin_Not_Sun_Exposed_Suprapubic | 101 | rs12720356 | rs142770866 | 1.27E-05 | 3.06E-05 | 1.78E-03 | 2.29E-03 | 1.00 |
| UC | Skin_Sun_Exposed_Lower_leg | 128 | rs34585310 | rs11668623 | 9.15E-55 | 5.14E-38 | 3.58E-19 | 1.82E-02 | 0.98 |
| IBD | Skin_Sun_Exposed_Lower_leg | 128 | rs281414 | rs281415 | 2.78E-42 | 1.08E-17 | 1.24E-27 | 2.81E-03 | 1.00 |
| IBD | Artery_Tibial | 107 | rs395782 | rs281420 | 2.65E-22 | 3.04E-15 | 5.06E-10 | 3.83E-03 | 1.00 |
| IBD | Lung | 30 | rs12720270 | rs74179926 | 6.41E-26 | 2.64E-06 | 1.65E-22 | 4.82E-03 | 1.00 |
| IBD | Thyroid | 122 | rs280525 | rs280519 | 7.44E-48 | 2.10E-18 | 2.61E-32 | 5.39E-03 | 0.99 |
| IBD | Esophagus_Gastroesophageal_Junction | 76 | rs1352426 | rs1387025 | 4.10E-54 | 3.66E-06 | 1.14E-50 | 8.17E-03 | 0.99 |
| IBD | Esophagus_Muscularis | 72 | rs8101473 | rs281422 | 1.25E-157 | 8.68E-157 | 4.23E-03 | 2.74E-02 | 0.97 |
| Psoriasis | Testis | 22 | rs12983921 | rs281427 | 1.25E-04 | 6.28E-06 | 9.06E-02 | 2.73E-03 | 0.91 |
| SLE | Thyroid | 119 | rs35251378 | rs11085725 | 5.75E-29 | 3.62E-22 | 9.54E-10 | 4.01E-03 | 1.00 |
| SLE | Adipose_Visceral_Omentum | 64 | rs35251378 | rs11085725 | 1.05E-09 | 3.74E-07 | 1.68E-05 | 4.00E-03 | 1.00 |
| SLE | Skin_Not_Sun_Exposed_Suprapubic | 101 | rs12972990 | rs150434441 | 0.00E+00 | 0.00E+00 | 6.49E-209 | 4.21E-03 | 1.00 |
| SLE | Breast_Mammary_Tissue | 112 | rs35251378 | rs11085725 | 2.41E-66 | 2.17E-41 | 7.15E-28 | 4.46E-03 | 1.00 |
| SLE | Colon_Sigmoid | 107 | rs35251378 | rs11085725 | 5.81E-16 | 3.86E-10 | 1.41E-08 | 7.41E-03 | 0.99 |
| SLE | Whole_Blood | 165 | rs78064630 | rs75231016 | 2.54E-12 | 5.69E-11 | 4.81E-04 | 8.81E-03 | 0.99 |
| SLE | Stomach | 13 | rs35251378 | rs2304256 | 4.20E-16 | 2.17E-10 | 2.27E-08 | 9.78E-03 | 0.99 |
| SLE | Adipose_Subcutaneous | 150 | rs35251378 | rs34725611 | 1.36E-51 | 1.25E-44 | 1.44E-09 | 1.12E-02 | 0.99 |
| T1D | Adipose_Visceral_Omentum | 70 | rs35251378 | rs11085725 | 0.00E+00 | 1.05E-155 | 6.04E-179 | 5.20E-03 | 0.99 |
| T1D | Lung | 31 | rs280519 | rs280526 | 1.87E-243 | 2.75E-163 | 6.46E-82 | 9.30E-02 | 0.91 |

CD, Crohn's Disease; IBD, inflammatory bowel disease; SLE, systemic lupus erythematosus; SNP, single nucleotide polymorphism; T1D, type 1 diabetes; UC, ulcerative colitis.

**Supplementary Table 12**. Associations of additional increase in minor (C) allele of rs34536443 with levels of 237 biomarkers.

| **Biomarker** | **Category** | **Sample size** | **Unit** | **Beta** | **SE** | **P** |
| --- | --- | --- | --- | --- | --- | --- |
| IGF-1 | serum/urine biochemistry | 342439 | mmol/l | -0.272 | 0.032 | 3.56E-17 |
| Plateletcrit | hematological traits | 408228 | % | -0.068 | 0.009 | 2.95E-15 |
| Lymphocyte count | hematological traits | 408228 | per nL | 0.063 | 0.008 | 7.58E-14 |
| Platelet count | hematological traits | 408228 | per nL | -0.061 | 0.009 | 7.28E-13 |
| Cystatin C | serum/urine biochemistry | 344264 | mmol/l | -0.005 | 0.001 | 1.04E-08 |
| Lymphocyte percentage of white cells | hematological traits | 408228 | % | 0.044 | 0.008 | 1.55E-07 |
| Eosinophil percentage of white cells | hematological traits | 408228 | % | -0.036 | 0.008 | 1.45E-05 |
| Sum neutrophil eosinophil counts | hematological traits | 408228 | per nL | -0.036 | 0.008 | 1.45E-05 |
| Aspartate aminotransferase | serum/urine biochemistry | 342990 | mmol/l | -0.264 | 0.062 | 1.89E-05 |
| White blood cell count | hematological traits | 408228 | per nL | 0.032 | 0.008 | 1.48E-04 |
| SHBG | serum/urine biochemistry | 312215 | mmol/l | -0.58 | 0.156 | 1.96E-04 |
| Cholesterol | serum lipids/metabolites | 344278 | SD | 0.024 | 0.006 | 2.74E-04 |
| Alkaline phosphatase | serum/urine biochemistry | 344292 | mmol/l | -0.533 | 0.153 | 4.89E-04 |
| IL18 | cytokines/growth factors | 3636 | SD | -0.273 | 0.079 | 6.45E-04 |
| Eosinophil percentage of granulocytes | hematological traits | 408228 | % | -0.028 | 0.008 | 9.43E-04 |
| Neutrophil percentage of white cells | hematological traits | 408228 | % | -0.027 | 0.008 | 1.03E-03 |
| Neutrophil percentage of granulocytes | hematological traits | 408228 | % | 0.027 | 0.008 | 0.002 |
| Rheumatoid factor | serum/urine biochemistry | 30565 | mmol/l | -1.214 | 0.393 | 0.002 |
| Eosinophil count | hematological traits | 408228 | per nL | -0.025 | 0.008 | 0.003 |
| Sum eosinophil basophil counts | hematological traits | 408228 | per nL | -0.024 | 0.008 | 0.004 |
| Basophil percentage of white cells | hematological traits | 408228 | % | -0.023 | 0.008 | 0.005 |
| SCGFb | cytokines/growth factors | 3682 | SD | -0.201 | 0.077 | 0.009 |
| MCSF | cytokines/growth factors | 840 | SD | -0.227 | 0.093 | 0.01 |
| Mean corpuscular volume | hematological traits | 408228 | fL | 0.018 | 0.008 | 0.027 |
| TRAIL | cytokines/growth factors | 8186 | SD | 0.101 | 0.049 | 0.031 |
| Mono unsaturated fatty acids | serum lipids/metabolites | 13531 | SD | 0.086 | 0.041 | 0.037 |
| Mean corpuscular hemoglobin | hematological traits | 408228 | pg | 0.017 | 0.008 | 0.038 |
| Red blood cell count | hematological traits | 408228 | per pL | -0.017 | 0.008 | 0.044 |
| Histidine | serum lipids/metabolites | 19238 | SD | 0.065 | 0.034 | 0.056 |
| Omega 7 and 9 and saturated fatty acids | serum lipids/metabolites | 13502 | SD | 0.077 | 0.041 | 0.062 |
| Ratio of bis allylic bonds to double bonds in lipids | serum lipids/metabolites | 13520 | SD | -0.077 | 0.041 | 0.064 |
| Alanine aminotransferase | serum/urine biochemistry | 344136 | mmol/l | -0.139 | 0.08 | 0.08 |
| Glutamine | serum lipids/metabolites | 24457 | SD | 0.053 | 0.031 | 0.084 |
| MCP3 | cytokines/growth factors | 843 | SD | -0.23 | 0.135 | 0.088 |
| Glycerol | serum lipids/metabolites | 20232 | SD | 0.056 | 0.034 | 0.096 |
| Fasting glucose | glycemic traits | 150080 | mmol/L | 0.011 | 0.006 | 0.097 |
| Total bilirubin | serum/urine biochemistry | 342829 | mmol/l | -0.041 | 0.025 | 0.108 |
| Total protein | serum/urine biochemistry | 314921 | mmol/l | -0.04 | 0.025 | 0.11 |
| MIG | cytokines/growth factors | 3685 | SD | -0.123 | 0.077 | 0.11 |
| Basophil percentage of granulocytes | hematological traits | 408228 | % | -0.013 | 0.008 | 0.115 |
| Direct bilirubin | serum/urine biochemistry | 292933 | mmol/l | -0.008 | 0.005 | 0.12 |
| GROa | cytokines/growth factors | 3505 | SD | -0.127 | 0.08 | 0.12 |
| IL8 | cytokines/growth factors | 3526 | SD | -0.12 | 0.08 | 0.12 |
| IL2ra | cytokines/growth factors | 3677 | SD | -0.123 | 0.077 | 0.121 |
| Total fatty acids | serum lipids/metabolites | 13501 | SD | 0.063 | 0.041 | 0.124 |
| Red cell distribution width | hematological traits | 408228 | fL | -0.013 | 0.008 | 0.125 |
| Fasting insulin | glycemic traits | 109731 | pmol/L | -0.007 | 0.007 | 0.127 |
| MIP1a | cytokines/growth factors | 3522 | SD | -0.116 | 0.08 | 0.129 |
| Urate | serum/urine biochemistry | 343836 | mmol/l | 0.609 | 0.402 | 0.13 |
| Glucose | serum lipids/metabolites | 24675 | SD | 0.046 | 0.03 | 0.134 |
| Glycine | serum lipids/metabolites | 18731 | SD | -0.05 | 0.035 | 0.154 |
| Ratio of bis allylic bonds to total fatty acids in lipids | serum lipids/metabolites | 13166 | SD | -0.059 | 0.042 | 0.157 |
| Monocyte percentage of white cells | hematological traits | 408228 | % | -0.012 | 0.008 | 0.162 |
| Tyrosine | serum lipids/metabolites | 24920 | SD | 0.042 | 0.03 | 0.169 |
| Neutrophil count | hematological traits | 408228 | per nL | 0.011 | 0.008 | 0.176 |
| VEGF | cytokines/growth factors | 7118 | SD | 0.075 | 0.053 | 0.178 |
| IL1ra | cytokines/growth factors | 3638 | SD | -0.102 | 0.078 | 0.187 |
| Lactate | serum lipids/metabolites | 24868 | SD | -0.039 | 0.03 | 0.198 |
| IFNg | cytokines/growth factors | 7701 | SD | 0.065 | 0.051 | 0.208 |
| Fatty acid length | serum lipids/metabolites | 13471 | SD | -0.052 | 0.041 | 0.209 |
| Sum basophil neutrophil counts | hematological traits | 408228 | per nL | 0.01 | 0.008 | 0.221 |
| Leucine | serum lipids/metabolites | 24723 | SD | 0.037 | 0.03 | 0.229 |
| Platelet distribution width | hematological traits | 408228 | fL | 0.01 | 0.008 | 0.233 |
| IL7 | cytokines/growth factors | 3409 | SD | -0.09 | 0.08 | 0.238 |
| Alanine | serum lipids/metabolites | 24792 | SD | 0.035 | 0.03 | 0.249 |
| BNGF | cytokines/growth factors | 3531 | SD | -0.082 | 0.078 | 0.25 |
| Esterified cholesterol | serum lipids/metabolites | 13493 | SD | 0.047 | 0.041 | 0.253 |
| DBP | hemodynamic traits | 317756 | SD | 0.007 | 0.006 | 0.255 |
| Microalbumin in urine | serum/urine biochemistry | 108706 | mmol/l | -0.012 | 0.01 | 0.269 |
| IP10 | cytokines/growth factors | 3685 | SD | -0.086 | 0.076 | 0.27 |
| IL4 | cytokines/growth factors | 8124 | SD | 0.056 | 0.049 | 0.273 |
| CH2 groups to double bonds ratio | serum lipids/metabolites | 13529 | SD | 0.043 | 0.041 | 0.299 |
| Myeloid white cell count | hematological traits | 408228 | per nL | 0.009 | 0.008 | 0.31 |
| Immature fraction of reticulocytes | hematological traits | 408228 | NA | 0.008 | 0.008 | 0.314 |
| Creatinine (enzymatic) in urine | serum/urine biochemistry | 327525 | mmol/l | 0.006 | 0.006 | 0.325 |
| Albumin | serum/urine biochemistry | 315268 | mmol/l | 0.016 | 0.016 | 0.326 |
| IL12p70 | cytokines/growth factors | 8270 | SD | 0.047 | 0.048 | 0.345 |
| Granulocyte count | hematological traits | 408228 | per nL | 0.008 | 0.008 | 0.35 |
| C-reactive protein | serum/urine biochemistry | 343524 | mmol/l | 0.024 | 0.026 | 0.355 |
| Lipoprotein A | serum lipids/metabolites | 13523 | SD | 0.038 | 0.041 | 0.358 |
| GCSF | cytokines/growth factors | 7904 | SD | 0.05 | 0.05 | 0.361 |
| Basophil count | hematological traits | 408228 | per nL | -0.007 | 0.008 | 0.381 |
| Creatinine | serum/urine biochemistry | 344104 | mmol/l | 0.08 | 0.092 | 0.386 |
| IL2 | cytokines/growth factors | 3475 | SD | -0.066 | 0.079 | 0.396 |
| FGFb | cytokines/growth factors | 7565 | SD | -0.036 | 0.05 | 0.398 |
| VLDL diameter | serum lipids/metabolites | 19267 | SD | 0.028 | 0.034 | 0.413 |
| Phosphatidylcholine and other cholines | serum lipids/metabolites | 13538 | SD | 0.032 | 0.041 | 0.434 |
| Phosphate | serum/urine biochemistry | 314658 | mmol/l | -0.001 | 0.001 | 0.442 |
| Total phosphoglycerides | serum lipids/metabolites | 13515 | SD | 0.031 | 0.041 | 0.447 |
| Mean platelet volume | hematological traits | 408228 | fL | 0.006 | 0.008 | 0.449 |
| Monocyte count | hematological traits | 408228 | per nL | 0.006 | 0.008 | 0.449 |
| IL5 | cytokines/growth factors | 3364 | SD | -0.052 | 0.081 | 0.462 |
| Hematocrit | hematological traits | 408228 | % | -0.006 | 0.008 | 0.471 |
| Phenylalanine | serum lipids/metabolites | 22658 | SD | 0.022 | 0.031 | 0.479 |
| HGF | cytokines/growth factors | 8292 | SD | -0.031 | 0.048 | 0.486 |
| Double bonds in fatty acids | serum lipids/metabolites | 15724 | SD | -0.026 | 0.037 | 0.487 |
| Creatinine | serum lipids/metabolites | 24805 | SD | -0.021 | 0.03 | 0.499 |
| 2 hour fasting glucose | glycemic traits | 42731 | mmol/L | -0.021 | 0.028 | 0.503 |
| Reticulocyte count | hematological traits | 408228 | pL | -0.006 | 0.008 | 0.505 |
| Pyruvate | serum lipids/metabolites | 24751 | SD | -0.02 | 0.03 | 0.505 |
| MCP1 | cytokines/growth factors | 8293 | SD | -0.026 | 0.048 | 0.52 |
| Other polyunsaturated fatty acids than 18:2 | serum lipids/metabolites | 13545 | SD | 0.026 | 0.041 | 0.521 |
| Mean corpuscular hemoglobin concentration | hematological traits | 408228 | g/dL | 0.005 | 0.008 | 0.522 |
| Omega 6 fatty acids | serum lipids/metabolites | 13502 | SD | 0.026 | 0.041 | 0.53 |
| Glycoprotein acetyls | serum lipids/metabolites | 19264 | SD | -0.021 | 0.034 | 0.531 |
| LDL diameter | serum lipids/metabolites | 19267 | SD | -0.021 | 0.034 | 0.533 |
| Triglycerides in very large VLDL | serum lipids/metabolites | 19153 | SD | 0.022 | 0.034 | 0.534 |
| Concentration of very large VLDL particles | serum lipids/metabolites | 15293 | SD | 0.025 | 0.039 | 0.535 |
| Total lipids in very large VLDL | serum lipids/metabolites | 19267 | SD | 0.02 | 0.034 | 0.556 |
| MIP1b | cytokines/growth factors | 8243 | SD | -0.025 | 0.048 | 0.557 |
| Total cholesterol in very large HDL | serum lipids/metabolites | 21534 | SD | 0.019 | 0.032 | 0.559 |
| Granulocyte percentage of myeloid white cells | hematological traits | 408228 | % | -0.005 | 0.008 | 0.582 |
| IL17 | cytokines/growth factors | 7760 | SD | 0.035 | 0.05 | 0.584 |
| Free cholesterol in medium VLDL | serum lipids/metabolites | 21234 | SD | 0.018 | 0.033 | 0.585 |
| Free cholesterol in very large HDL | serum lipids/metabolites | 21536 | SD | 0.017 | 0.032 | 0.603 |
| Free cholesterol in IDL | serum lipids/metabolites | 21553 | SD | -0.017 | 0.032 | 0.614 |
| IL1b | cytokines/growth factors | 3309 | SD | -0.119 | 0.062 | 0.619 |
| Phospholipids in small VLDL | serum lipids/metabolites | 21545 | SD | 0.016 | 0.032 | 0.627 |
| Cholesterol esters in medium HDL | serum lipids/metabolites | 19267 | SD | -0.016 | 0.034 | 0.632 |
| Triglycerides in small VLDL | serum lipids/metabolites | 21552 | SD | 0.015 | 0.032 | 0.653 |
| Free cholesterol | serum lipids/metabolites | 13493 | SD | 0.019 | 0.041 | 0.654 |
| Total lipids in medium VLDL | serum lipids/metabolites | 19267 | SD | 0.015 | 0.034 | 0.657 |
| Oestradiol | serum/urine biochemistry | 54498 | mmol/l | 2.666 | 6.112 | 0.663 |
| Phospholipids in medium HDL | serum lipids/metabolites | 21552 | SD | -0.014 | 0.032 | 0.665 |
| Total cholesterol in medium HDL | serum lipids/metabolites | 21552 | SD | -0.014 | 0.032 | 0.665 |
| Concentration of IDL particles | serum lipids/metabolites | 19267 | SD | -0.015 | 0.034 | 0.665 |
| Free cholesterol in small VLDL | serum lipids/metabolites | 21553 | SD | 0.014 | 0.032 | 0.667 |
| Total lipids in IDL | serum lipids/metabolites | 19267 | SD | -0.015 | 0.034 | 0.67 |
| IL16 | cytokines/growth factors | 3483 | SD | -0.033 | 0.078 | 0.67 |
| Cholesterol esters in medium VLDL | serum lipids/metabolites | 19267 | SD | 0.014 | 0.034 | 0.673 |
| CTACK | cytokines/growth factors | 3631 | SD | -0.033 | 0.078 | 0.675 |
| Testosterone | serum/urine biochemistry | 312102 | mmol/l | 0.007 | 0.017 | 0.681 |
| CH2 groups in fatty acids | serum lipids/metabolites | 19017 | SD | 0.014 | 0.035 | 0.682 |
| Cholesterol esters in very large HDL | serum lipids/metabolites | 19267 | SD | 0.014 | 0.034 | 0.687 |
| IL10 | cytokines/growth factors | 7681 | SD | 0.02 | 0.05 | 0.69 |
| Free cholesterol in large VLDL | serum lipids/metabolites | 21232 | SD | 0.013 | 0.033 | 0.691 |
| RANTES | cytokines/growth factors | 3421 | SD | -0.03 | 0.08 | 0.691 |
| Phospholipids in IDL | serum lipids/metabolites | 21553 | SD | -0.013 | 0.032 | 0.694 |
| Vitamin D | serum/urine biochemistry | 329247 | mmol/l | -0.05 | 0.126 | 0.694 |
| Glucose | glycemic traits | 12321875 | mmol/L | 0.004 | 0.01 | 0.7 |
| Total cholesterol in IDL | serum lipids/metabolites | 19267 | SD | -0.013 | 0.034 | 0.7 |
| Triglycerides in very large HDL | serum lipids/metabolites | 21530 | SD | -0.012 | 0.032 | 0.704 |
| Triglycerides in large VLDL | serum lipids/metabolites | 21233 | SD | 0.012 | 0.033 | 0.705 |
| Concentration of very large HDL particles | serum lipids/metabolites | 19267 | SD | -0.013 | 0.034 | 0.705 |
| Total lipids in small VLDL | serum lipids/metabolites | 19267 | SD | 0.013 | 0.034 | 0.707 |
| Total cholesterol in small VLDL | serum lipids/metabolites | 21551 | SD | 0.012 | 0.032 | 0.71 |
| Triglycerides in medium VLDL | serum lipids/metabolites | 21235 | SD | 0.012 | 0.033 | 0.712 |
| Total cholesterol in medium VLDL | serum lipids/metabolites | 21545 | SD | 0.012 | 0.032 | 0.715 |
| Valine | serum lipids/metabolites | 24894 | SD | 0.011 | 0.03 | 0.717 |
| Concentration of small VLDL particles | serum lipids/metabolites | 19267 | SD | 0.012 | 0.034 | 0.717 |
| Total cholesterol in large VLDL | serum lipids/metabolites | 21229 | SD | 0.012 | 0.033 | 0.719 |
| Total lipids in small HDL | serum lipids/metabolites | 19267 | SD | -0.012 | 0.034 | 0.72 |
| Triglycerides in small HDL | serum lipids/metabolites | 21552 | SD | -0.012 | 0.032 | 0.72 |
| Total lipids in medium HDL | serum lipids/metabolites | 19267 | SD | -0.012 | 0.034 | 0.726 |
| Urea | serum lipids/metabolites | 18808 | SD | 0.012 | 0.034 | 0.727 |
| Phospholipids in very small VLDL | serum lipids/metabolites | 19267 | SD | -0.012 | 0.034 | 0.732 |
| Acetate | serum lipids/metabolites | 24742 | SD | 0.01 | 0.03 | 0.732 |
| Citrate | serum lipids/metabolites | 24764 | SD | -0.01 | 0.03 | 0.736 |
| Total lipids in large VLDL | serum lipids/metabolites | 18954 | SD | 0.012 | 0.034 | 0.736 |
| Isoleucine | serum lipids/metabolites | 24771 | SD | 0.01 | 0.03 | 0.74 |
| Free cholesterol in large LDL | serum lipids/metabolites | 21549 | SD | -0.01 | 0.032 | 0.754 |
| Concentration of large LDL particles | serum lipids/metabolites | 19267 | SD | -0.011 | 0.034 | 0.755 |
| Apolipoprotein A | serum lipids/metabolites | 18400 | SD | 0.011 | 0.034 | 0.756 |
| Serum total triglycerides | serum lipids/metabolites | 21539 | SD | 0.01 | 0.032 | 0.758 |
| Concentration of medium VLDL particles | serum lipids/metabolites | 19267 | SD | 0.01 | 0.034 | 0.76 |
| Concentration of medium HDL particles | serum lipids/metabolites | 19267 | SD | -0.01 | 0.034 | 0.763 |
| Phospholipids in medium VLDL | serum lipids/metabolites | 21234 | SD | 0.01 | 0.033 | 0.766 |
| Concentration of chylomicrons and extremely large VLDL particles | serum lipids/metabolites | 18954 | SD | -0.01 | 0.034 | 0.77 |
| Omega 3 fatty acids | serum lipids/metabolites | 13540 | SD | 0.012 | 0.041 | 0.772 |
| Phospholipids in chylomicrons and extremely large VLDL | serum lipids/metabolites | 21536 | SD | -0.009 | 0.032 | 0.772 |
| Apolipoprotein B | serum lipids/metabolites | 20686 | SD | -0.01 | 0.033 | 0.773 |
| Total cholesterol in medium LDL | serum lipids/metabolites | 21553 | SD | -0.009 | 0.032 | 0.775 |
| Total lipids in large LDL | serum lipids/metabolites | 19267 | SD | -0.01 | 0.034 | 0.779 |
| Hemoglobin concentration | hematological traits | 408228 | g/dL | -0.002 | 0.008 | 0.782 |
| Pulse rate | hemodynamic traits | 317756 | SD | -0.002 | 0.006 | 0.784 |
| EOTAXIN | cytokines/growth factors | 8153 | SD | -0.009 | 0.049 | 0.792 |
| Triglycerides in IDL | serum lipids/metabolites | 19267 | SD | -0.009 | 0.034 | 0.793 |
| IL6 | cytokines/growth factors | 8189 | SD | 0.016 | 0.048 | 0.797 |
| PDGFbb | cytokines/growth factors | 8293 | SD | -0.009 | 0.048 | 0.799 |
| 22:6 docosahexaenoic acid (DHA) | serum lipids/metabolites | 13496 | SD | 0.01 | 0.041 | 0.801 |
| Free cholesterol in medium HDL | serum lipids/metabolites | 21553 | SD | -0.008 | 0.032 | 0.807 |
| High light scatter percentage of red cells | hematological traits | 408228 | % | 0.002 | 0.008 | 0.813 |
| MIF | cytokines/growth factors | 3494 | SD | 0.02 | 0.082 | 0.818 |
| Urea | serum/urine biochemistry | 344052 | mmol/l | 0.002 | 0.008 | 0.82 |
| HDL diameter | serum lipids/metabolites | 19267 | SD | -0.007 | 0.034 | 0.828 |
| Triglycerides in chylomicrons and extremely large VLDL | serum lipids/metabolites | 21534 | SD | 0.007 | 0.032 | 0.837 |
| IL13 | cytokines/growth factors | 3557 | SD | 0.021 | 0.078 | 0.84 |
| SBP | hemodynamic traits | 317754 | SD | 0.001 | 0.006 | 0.843 |
| Triglycerides in very small VLDL | serum lipids/metabolites | 19267 | SD | 0.007 | 0.034 | 0.843 |
| Total lipids in very large HDL | serum lipids/metabolites | 19267 | SD | 0.007 | 0.034 | 0.843 |
| Total lipids in small LDL | serum lipids/metabolites | 19267 | SD | -0.006 | 0.034 | 0.854 |
| Calcium | serum/urine biochemistry | 315153 | mmol/l | 0 | 0.001 | 0.859 |
| Concentration of small HDL particles | serum lipids/metabolites | 19267 | SD | -0.006 | 0.034 | 0.86 |
| Concentration of large VLDL particles | serum lipids/metabolites | 18954 | SD | -0.006 | 0.034 | 0.865 |
| Total lipids in chylomicrons and extremely large VLDL | serum lipids/metabolites | 18954 | SD | -0.006 | 0.034 | 0.869 |
| Glycated haemoglobin (HbA1c) | glycemic traits | 116418 | % | 0.001 | 0.004 | 0.873 |
| Reticulocyte fraction of red cells | hematological traits | 408228 | % | -0.001 | 0.008 | 0.874 |
| Concentration of medium LDL particles | serum lipids/metabolites | 19267 | SD | -0.005 | 0.034 | 0.877 |
| Total lipids in medium LDL | serum lipids/metabolites | 19267 | SD | -0.005 | 0.034 | 0.881 |
| Concentration of large HDL particles | serum lipids/metabolites | 19267 | SD | -0.005 | 0.034 | 0.883 |
| Phospholipids in large HDL | serum lipids/metabolites | 19267 | SD | -0.005 | 0.034 | 0.884 |
| Gamma glutamyltransferase | serum/urine biochemistry | 344104 | mmol/l | 0.036 | 0.245 | 0.884 |
| Cholesterol esters in large LDL | serum lipids/metabolites | 19267 | SD | -0.005 | 0.034 | 0.89 |
| Phospholipids in large VLDL | serum lipids/metabolites | 21233 | SD | 0.005 | 0.033 | 0.89 |
| Total cholesterol in large HDL | serum lipids/metabolites | 21552 | SD | 0.005 | 0.032 | 0.89 |
| Concentration of very small VLDL particles | serum lipids/metabolites | 19267 | SD | 0.004 | 0.034 | 0.902 |
| Sphingomyelins | serum lipids/metabolites | 13474 | SD | 0.005 | 0.041 | 0.907 |
| Cholesterol esters in medium LDL | serum lipids/metabolites | 19267 | SD | -0.004 | 0.034 | 0.907 |
| Total lipids in very small VLDL | serum lipids/metabolites | 19267 | SD | 0.004 | 0.034 | 0.914 |
| Total cholesterol in large LDL | serum lipids/metabolites | 21546 | SD | -0.003 | 0.032 | 0.919 |
| Potassium in urine | serum/urine biochemistry | 326816 | mmol/l | 0.001 | 0.006 | 0.924 |
| Cholesterol esters in large VLDL | serum lipids/metabolites | 18954 | SD | -0.003 | 0.034 | 0.926 |
| Total cholesterol in small LDL | serum lipids/metabolites | 21550 | SD | -0.003 | 0.032 | 0.928 |
| Total lipids in large HDL | serum lipids/metabolites | 19267 | SD | -0.003 | 0.034 | 0.934 |
| IL9 | cytokines/growth factors | 3634 | SD | -0.002 | 0.078 | 0.935 |
| Phospholipids in large LDL | serum lipids/metabolites | 21544 | SD | -0.003 | 0.032 | 0.939 |
| SCF | cytokines/growth factors | 8290 | SD | 0.004 | 0.048 | 0.941 |
| Cholesterol esters in large HDL | serum lipids/metabolites | 19267 | SD | 0.002 | 0.034 | 0.943 |
| Sodium in urine | serum/urine biochemistry | 326831 | mmol/l | 0 | 0.006 | 0.945 |
| HDL cholesterol | serum lipids/metabolites | 21549 | SD | -0.001 | 0.032 | 0.965 |
| High light scatter reticulocyte count | hematological traits | 408228 | per pL | 0 | 0.008 | 0.968 |
| TNF-a | cytokines/growth factors | 3454 | SD | 0.002 | 0.08 | 0.969 |
| Phospholipids in very large HDL | serum lipids/metabolites | 19267 | SD | 0.001 | 0.034 | 0.972 |
| Phospholipids in medium LDL | serum lipids/metabolites | 21552 | SD | 0.001 | 0.032 | 0.975 |
| Phospholipids in very large VLDL | serum lipids/metabolites | 21231 | SD | -0.001 | 0.033 | 0.983 |
| Serum total cholesterol | serum lipids/metabolites | 21485 | SD | -0.001 | 0.032 | 0.984 |
| Concentration of small LDL particles | serum lipids/metabolites | 19267 | SD | 0.001 | 0.034 | 0.984 |
| LDL direct | serum lipids/metabolites | 21553 | SD | 0.001 | 0.032 | 0.988 |
| SDF1a | cytokines/growth factors | 5998 | SD | -0.02 | 0.05 | 0.988 |
| Free cholesterol in large HDL | serum lipids/metabolites | 21553 | SD | 0 | 0.032 | 0.991 |
| Acetoacetate | serum lipids/metabolites | 19257 | SD | 0 | 0.034 | 0.991 |

**Supplementary Table 13. Mediation analysis for selected biomarkers in the UK Biobank**

| **Biomarker** | **Phenotypes** | **ACME effect** | **ACME LB** | **ACME UB** | **ACME p** | **ADE effect** | **ADE LB** | **ADE UB** | **ADE p** |
| --- | --- | --- | --- | --- | --- | --- | --- | --- | --- |
| Cystatin C | Hypothyroidism NOS | -1.00E-04 | -1.56E-04 | -5.55E-05 | 4.65E-04 | -8.56E-03 | -1.11E-02 | -5.86E-03 | 3.97E-11 |
| Cystatin C | Psoriasis | 3.46E-07 | -9.75E-06 | 1.19E-05 | 9.46E-01 | -3.80E-03 | -4.61E-03 | -2.79E-03 | 3.75E-20 |
| Cystatin C | Psoriasis and related disorders | -8.28E-07 | -1.34E-05 | 1.03E-05 | 8.97E-01 | -3.94E-03 | -4.93E-03 | -2.81E-03 | 6.17E-15 |
| Cystatin C | Hypothyroidism | -1.05E-04 | -1.45E-04 | -7.23E-05 | 2.68E-07 | -8.20E-03 | -1.02E-02 | -5.85E-03 | 9.28E-16 |
| Cystatin C | Rheumatoid arthritis and other inflammatory polyarthropathies | -1.23E-05 | -2.40E-05 | -2.34E-06 | 3.93E-02 | -3.80E-03 | -4.97E-03 | -2.55E-03 | 1.94E-10 |
| Cystatin C | Rheumatoid arthritis | -9.22E-06 | -1.98E-05 | 1.68E-06 | 8.76E-02 | -3.42E-03 | -4.41E-03 | -2.17E-03 | 1.28E-11 |
| Cystatin C | Psoriasis vulgaris | -6.25E-09 | -1.07E-05 | 6.87E-06 | 9.99E-01 | -3.00E-03 | -3.84E-03 | -1.98E-03 | 2.56E-12 |
| Cystatin C | Inflammatory bowel disease and other gastroenteritis and colitis | 7.28E-06 | -4.98E-06 | 2.19E-05 | 2.44E-01 | -3.18E-03 | -4.61E-03 | -1.92E-03 | 1.31E-05 |
| Cystatin C | Psoriatic arthropathy | 1.21E-06 | -3.88E-06 | 6.84E-06 | 6.41E-01 | -1.42E-03 | -1.83E-03 | -8.43E-04 | 1.13E-11 |
| Cystatin C | Congenital deformities of feet | 6.47E-07 | -2.16E-06 | 2.71E-06 | 6.51E-01 | 7.43E-04 | 3.38E-04 | 1.28E-03 | 3.24E-04 |
| Cystatin C | Ulcerative colitis | 1.39E-05 | 1.95E-06 | 2.76E-05 | 2.26E-02 | -2.49E-03 | -3.43E-03 | -1.24E-03 | 2.08E-07 |
| Cystatin C | Chronic hepatitis | -5.29E-06 | -8.70E-06 | -1.73E-06 | 2.36E-03 | -7.03E-04 | -9.22E-04 | -4.48E-04 | 3.14E-10 |
| Cystatin C | Congenital anomalies of stomach | -1.30E-06 | -4.68E-06 | 4.61E-07 | 4.51E-01 | 2.83E-04 | 2.30E-05 | 6.64E-04 | 3.29E-02 |
| Cystatin C | Type 1 diabetes | -2.48E-05 | -3.71E-05 | -1.39E-05 | 7.75E-05 | -2.02E-03 | -2.98E-03 | -7.07E-04 | 3.72E-05 |
| Cystatin C | Celiac disease | -4.75E-05 | -7.12E-05 | -2.79E-05 | 8.56E-05 | -1.71E-03 | -2.42E-03 | -1.06E-03 | 2.35E-06 |
| Cystatin C | Diffuse diseases of connective tissue | -8.64E-06 | -1.65E-05 | -2.39E-06 | 3.12E-02 | -1.29E-03 | -1.85E-03 | -6.95E-04 | 6.33E-06 |
| IGF-1 | Hypothyroidism NOS | 5.86E-05 | 2.96E-05 | 9.45E-05 | 7.48E-05 | -8.89E-03 | -1.11E-02 | -7.05E-03 | 3.16E-15 |
| IGF-1 | Psoriasis | 3.89E-06 | -9.71E-06 | 1.52E-05 | 5.75E-01 | -4.01E-03 | -4.81E-03 | -2.93E-03 | 8.83E-23 |
| IGF-1 | Psoriasis and related disorders | 2.43E-06 | -1.06E-05 | 1.62E-05 | 7.15E-01 | -3.96E-03 | -5.08E-03 | -3.14E-03 | 4.21E-12 |
| IGF-1 | Hypothyroidism | 5.69E-05 | 2.91E-05 | 9.03E-05 | 6.03E-05 | -8.19E-03 | -1.01E-02 | -6.08E-03 | 4.30E-17 |
| IGF-1 | Rheumatoid arthritis and other inflammatory polyarthropathies | -5.23E-06 | -1.91E-05 | 9.70E-06 | 4.60E-01 | -3.90E-03 | -5.07E-03 | -2.64E-03 | 6.43E-11 |
| IGF-1 | Rheumatoid arthritis | -1.73E-06 | -1.46E-05 | 1.25E-05 | 7.92E-01 | -3.33E-03 | -4.45E-03 | -2.27E-03 | 5.63E-09 |
| IGF-1 | Psoriasis vulgaris | 3.86E-06 | -6.77E-06 | 1.69E-05 | 4.77E-01 | -2.95E-03 | -3.79E-03 | -2.04E-03 | 5.85E-12 |
| IGF-1 | Inflammatory bowel disease and other gastroenteritis and colitis | -1.07E-05 | -2.88E-05 | 3.54E-06 | 2.47E-01 | -3.17E-03 | -4.69E-03 | -1.84E-03 | 4.36E-05 |
| IGF-1 | Psoriatic arthropathy | 6.63E-07 | -4.87E-06 | 6.85E-06 | 8.14E-01 | -1.44E-03 | -1.91E-03 | -8.68E-04 | 1.91E-09 |
| IGF-1 | Congenital deformities of feet | -6.34E-07 | -2.92E-06 | 2.29E-06 | 5.87E-01 | 7.20E-04 | 3.60E-04 | 1.21E-03 | 8.85E-05 |
| IGF-1 | Ulcerative colitis | -8.25E-06 | -2.18E-05 | 3.76E-06 | 2.33E-01 | -2.45E-03 | -3.61E-03 | -1.14E-03 | 3.48E-05 |
| IGF-1 | Chronic hepatitis | 6.95E-07 | -2.01E-06 | 4.56E-06 | 6.15E-01 | -7.51E-04 | -9.78E-04 | -5.11E-04 | 8.91E-11 |
| IGF-1 | Congenital anomalies of stomach | 3.02E-06 | -5.19E-08 | 1.35E-05 | 5.40E-02 | 3.18E-04 | 8.39E-05 | 1.13E-03 | 7.76E-03 |
| IGF-1 | Type 1 diabetes | 3.75E-06 | -6.68E-06 | 1.34E-05 | 4.81E-01 | -1.97E-03 | -2.67E-03 | -9.10E-04 | 3.47E-08 |
| IGF-1 | Celiac disease | 2.02E-05 | 1.02E-05 | 3.43E-05 | 7.52E-05 | -1.69E-03 | -2.64E-03 | -7.08E-04 | 4.89E-04 |
| IGF-1 | Diffuse diseases of connective tissue | 4.25E-06 | -3.27E-06 | 1.15E-05 | 2.68E-01 | -1.25E-03 | -1.82E-03 | -7.32E-04 | 1.72E-05 |
| SHBG | Hypothyroidism NOS | -4.58E-08 | -4.74E-06 | 5.06E-06 | 9.85E-01 | -8.69E-03 | -1.08E-02 | -6.19E-03 | 6.90E-16 |
| SHBG | Psoriasis | 2.45E-07 | -2.85E-06 | 3.79E-06 | 8.77E-01 | -3.93E-03 | -4.81E-03 | -2.88E-03 | 2.07E-18 |
| SHBG | Psoriasis and related disorders | 5.13E-07 | -2.21E-06 | 4.87E-06 | 7.12E-01 | -3.94E-03 | -4.74E-03 | -3.09E-03 | 4.77E-22 |
| SHBG | Hypothyroidism | -7.08E-08 | -4.42E-06 | 4.10E-06 | 9.75E-01 | -8.08E-03 | -1.06E-02 | -5.56E-03 | 3.29E-10 |
| SHBG | Rheumatoid arthritis and other inflammatory polyarthropathies | -8.88E-08 | -2.58E-06 | 1.88E-06 | 9.44E-01 | -3.76E-03 | -4.89E-03 | -2.55E-03 | 6.95E-11 |
| SHBG | Rheumatoid arthritis | -1.02E-07 | -2.59E-06 | 1.87E-06 | 9.36E-01 | -3.45E-03 | -4.51E-03 | -2.59E-03 | 1.78E-10 |
| SHBG | Psoriasis vulgaris | 1.86E-07 | -2.23E-06 | 2.58E-06 | 8.80E-01 | -2.97E-03 | -3.68E-03 | -1.92E-03 | 2.43E-16 |
| SHBG | Inflammatory bowel disease and other gastroenteritis and colitis | 1.15E-07 | -4.09E-06 | 3.94E-06 | 9.57E-01 | -3.24E-03 | -4.43E-03 | -1.68E-03 | 9.48E-08 |
| SHBG | Psoriatic arthropathy | -1.66E-07 | -1.70E-06 | 6.55E-07 | 8.32E-01 | -1.45E-03 | -1.91E-03 | -1.04E-03 | 6.48E-10 |
| SHBG | Congenital deformities of feet | 9.55E-09 | -9.41E-07 | 1.02E-06 | 9.84E-01 | 7.83E-04 | 3.19E-04 | 1.19E-03 | 9.41E-04 |
| SHBG | Ulcerative colitis | -1.76E-07 | -2.96E-06 | 2.05E-06 | 9.01E-01 | -2.44E-03 | -3.48E-03 | -1.20E-03 | 4.26E-06 |
| SHBG | Chronic hepatitis | 5.37E-08 | -6.72E-07 | 7.73E-07 | 8.85E-01 | -7.20E-04 | -9.36E-04 | -4.86E-04 | 6.43E-11 |
| SHBG | Congenital anomalies of stomach | -6.15E-08 | -7.78E-07 | 3.39E-07 | 8.66E-01 | 3.61E-04 | 8.84E-05 | 1.38E-03 | 9.44E-03 |
| SHBG | Type 1 diabetes | -1.13E-07 | -7.38E-06 | 7.65E-06 | 9.76E-01 | -1.99E-03 | -2.92E-03 | -1.06E-03 | 2.74E-05 |
| SHBG | Celiac disease | 7.33E-08 | -3.37E-06 | 2.11E-06 | 9.67E-01 | -1.69E-03 | -2.62E-03 | -7.66E-04 | 3.68E-04 |
| SHBG | Diffuse diseases of connective tissue | -3.49E-08 | -1.21E-06 | 1.01E-06 | 9.54E-01 | -1.31E-03 | -1.87E-03 | -5.66E-04 | 4.54E-06 |
| IL18 | Hypothyroidism NOS | -1.29E-06 | -8.48E-06 | 4.38E-06 | 7.25E-01 | -8.71E-03 | -1.10E-02 | -6.41E-03 | 9.00E-14 |
| IL18 | Psoriasis | -3.26E-06 | -1.01E-05 | 3.27E-06 | 3.50E-01 | -3.87E-03 | -4.70E-03 | -2.93E-03 | 6.32E-20 |
| IL18 | Psoriasis and related disorders | -3.08E-06 | -9.46E-06 | 2.78E-06 | 3.44E-01 | -3.84E-03 | -4.80E-03 | -2.85E-03 | 4.51E-15 |
| IL18 | Hypothyroidism | -1.27E-06 | -1.01E-05 | 4.69E-06 | 7.78E-01 | -8.25E-03 | -1.04E-02 | -5.49E-03 | 5.44E-14 |
| IL18 | Rheumatoid arthritis and other inflammatory polyarthropathies | 4.18E-08 | -2.68E-06 | 2.62E-06 | 9.76E-01 | -3.84E-03 | -5.25E-03 | -2.69E-03 | 9.40E-08 |
| IL18 | Rheumatoid arthritis | -1.96E-07 | -3.28E-06 | 2.98E-06 | 9.01E-01 | -3.45E-03 | -4.47E-03 | -2.22E-03 | 3.37E-11 |
| IL18 | Psoriasis vulgaris | -2.20E-06 | -8.77E-06 | 1.76E-06 | 5.12E-01 | -2.97E-03 | -3.61E-03 | -2.20E-03 | 9.40E-20 |
| IL18 | Inflammatory bowel disease and other gastroenteritis and colitis | -1.77E-06 | -8.75E-06 | 3.34E-06 | 6.19E-01 | -3.06E-03 | -4.46E-03 | -1.32E-03 | 1.84E-05 |
| IL18 | Psoriatic arthropathy | -1.44E-06 | -4.84E-06 | 8.42E-07 | 4.06E-01 | -1.44E-03 | -1.93E-03 | -8.98E-04 | 8.41E-09 |
| IL18 | Congenital deformities of feet | 1.35E-08 | -6.23E-07 | 9.12E-07 | 9.67E-01 | 7.09E-04 | 2.97E-04 | 1.20E-03 | 7.44E-04 |
| IL18 | Ulcerative colitis | -1.18E-06 | -7.24E-06 | 4.18E-06 | 7.03E-01 | -2.46E-03 | -3.63E-03 | -1.01E-03 | 3.77E-05 |
| IL18 | Chronic hepatitis | -4.58E-07 | -1.96E-06 | 3.52E-07 | 5.50E-01 | -7.39E-04 | -9.96E-04 | -4.71E-04 | 1.74E-08 |
| IL18 | Congenital anomalies of stomach | 1.24E-07 | -2.45E-07 | 6.27E-07 | 5.10E-01 | 2.72E-04 | 2.79E-05 | 5.98E-04 | 2.90E-02 |
| IL18 | Type 1 diabetes | 1.28E-06 | -7.15E-07 | 5.78E-06 | 2.09E-01 | -2.00E-03 | -2.87E-03 | -9.47E-04 | 6.61E-06 |
| IL18 | Celiac disease | -3.46E-07 | -3.77E-06 | 3.70E-06 | 8.43E-01 | -1.72E-03 | -2.67E-03 | -7.04E-04 | 3.87E-04 |
| IL18 | Diffuse diseases of connective tissue | -6.01E-07 | -3.10E-06 | 8.87E-07 | 6.37E-01 | -1.24E-03 | -1.77E-03 | -6.18E-04 | 4.53E-06 |

ACME, average causal mediation effects (indirect effect explained by mediator); ADE, average direct effects (direct effect explained by exposure); IGF-1, insulin-like growth factor 1; IL18, interleukin 18; LB, low bound of 95% confidence interval; SHBG, sex hormone binding globulin; UB, up bound of 95% confidence interval.

**Supplementary Table 14.** Characteristics of 21 included randomized controlled trails in systematic review

| **Study** | **Title** | **NCT number** | **Phase** | **Drug** | **Target** | **Condition** | **Gender** | **Age** | **Interventions** | **N** | **Primary endpoint** | **Secondary endpoint** | **Adverse events** |
| --- | --- | --- | --- | --- | --- | --- | --- | --- | --- | --- | --- | --- | --- |
| Armstrong  2021 | Efficacy and safety of deucravacitinib, an oral, selective tyrosine kinase 2 (tyk2) inhibitor, compared with placebo and apremilast in moderate to severe plaque psoriasis: Results from the phase 3 poetyk pso-1 study | NCT03624127 | 3 | BMS-986165 | TYK2 | Plaque Psoriasis | All | >=18 | 6mg QD, placebo, or Apremilast 30mg BID (2:1:1) | 666 | PASI 75 and sPGA 0/1 response at week 16 | Superiority versus apremilast | Nasopharyngitis, upper respiratory tract infection, headache, diarrhea, and nausea |
| Banfield  2018 | The Safety, Tolerability, Pharmacokinetics, and Pharmacodynamics of a TYK2/JAK1 Inhibitor (PF-06700841) in Healthy Subjects and Patients With Plaque Psoriasis | NCT02310750 | 1 | PF-06700841 | TYK2/JAK1 | Healthy participants/ Plaque Psoriasis | All | 18-65 | Healthy participants: SAD (1, 3, 10, 30, 100, and 200 mg once or placebo) and MAD (10, 30, 100, 175 mg QD or 50 mg BID or placebo); Patiants: 30, 100 mg QD or placebo | 96 | PK, PD parameters, and safety | Change from baseline in PASI score over time | Increased serum creatinine |
| Catlett  2021 | Molecular and clinical effects of selective tyrosine kinase 2 inhibition with deucravacitinib in psoriasis | NCT02931838 | 2 | BMS-986165 | TYK2 | Plaque Psoriasis | All | 18-70 | 3mg QOD, 3mg QD, 3mg BID, 6mg BID, 12mg QD or placebo (1:1:1:1:1:1) | 267 | Epidermal thickness and inflammatory cell infiltration; psoriasis-related differentially expressed genes; expression of genes in IL-23/IL-22 and Type1 IFN pathyway | Selectivity profiling versus JAK1-3 | - |
| Chimalakonda  2022 | Lack of Electrocardiographic Effects of Deucravacitinib in Healthy Subjects | NA | 1 | BMS-986165 | TYK2 | Healthy participants | All | 18-50 | 12, 36mg, placebo, or moxifloxacin | 157 | Placebo-corrected change from baseline for the QT interval corrected for heart rate using the Fridericia method | Electrocardiographic parameters | Headache |
| Danese 2022 | Efficacy and safety of deucravacitinib, an oral, selective tyrosine kinase 2 inhibitor, in patients with moderately-to-severely active Ulcerative Colitis: 12-week results from the Phase 2 LATTICEUC study | NCT03934216 | 2 | BMS-986165 | TYK2 | Ulcerative Colitis | All | 18-80 | 6mg BID or placebo (2:1） | 131 | Clinical remission evaluated by modified Mayo score at week 12 | Endoscopic response <=1 at week 12 | Rash, acne, and worsening of ulcerative colitis |
| Forman 2020 | TYK2/JAK1 Inhibitor PF-06700841 in Patients with Plaque Psoriasis: Phase IIa, Randomized, Double-Blind, Placebo-Controlled Trial | NCT02969018 | 2a | PF-06700841 | TYK2/JAK1 | Plaque Psoriasis | All | 18-75 | 4w incudtion: 30mg QD or 60mg QD or placebo (4:3:1); 8w maintenance: 10mg QD or 30mg QD or 100mg QW or placebo (2:2:2:2) | 212 | Change from baseline in PASI score at week 12 | Proportion of patients achieving 75% and 90% reduction from baseline PASI at week 12 | Nasopharyngitis, upper respiratory tract infection, and headache |
| Guttman-Yasskky 2021 | Ritlecitinib and brepocitinib demonstrate significant improvement in scalp alopecia areata biomarkers | NCT02974868 | 2a | PF-06700841 | TYK2/JAK1 | Alopecia Areata | All | 18-75 | 60 mgQD for 4 weeks, then 30 mgQD for 20 weeks or placebo (2:1) | 94 | Change in biomarkers in lesional scalp biopsy samples between baseline and weeks 12 and 24 | Correlations of changes in biomarkers and percent SALT score reduction at weeks 12 and 24 | - |
| King 2021 | A phase 2a randomized, placebo-controlled study to evaluate the efficacy and safety of the oral Janus kinase inhibitors ritlecitinib and brepocitinib in alopecia areata: 24-week results | NCT02974868 | 2a | PF-06700841 | TYK2/JAK1 | Alopecia Areata | All | 18-75 | 60 mgQD for 4 weeks, then 30 mgQD for 20 weeks or placebo (2:1) | 94 | Change from baseline in SALT score at week 24 | Proportion of patients achieving 30% improvement in SALT score at week 24 | Upper respiratory tract infection, nasopharyngitis, headache, acne, and nausea |
| Mease 2021 | Efficacy and safety of brepocitinib (tyrosine kinase 2/janus kinase 1 inhibitor) for the treatment of active psoriatic arthritis: Results from a phase 2b randomized controlled trial | NCT03963401 | 2b | PF-06700841 | TYK2/JAK1 | Psoriatic Arthritis | All | 18-75 | 10, 30, 60mg QD or placebo (1:2:2:2) | 218 | ACR-20 response at Week 16 | PASDAS, HAQ-DI, SF-36 PCS, and FACIT-F at week 16 | Infestious and infestations, neoplasms, benign,malifnant,and unspecified(soc) |
| Mease 2022 | Efficacy and safety of selective TYK2 inhibitor, deucravacitinib, in a phase II trial in psoriatic arthritis | NCT03881059 | 2 | BMS-986165 | TYK2 | Psoriatic Arthritis | All | >=18 | 6mg QD or 12mg QD or placebo for 16 weeks (1:1:1) | 203 | ACR-20 response at week 16 | Change from baseline in HAQ-DI score; PASI-75 response; and change from baseline in SF-36 PCS | Nasopharyngitis, upper respiratory tract infection, sinusitis, bronchitis, rash, diarrhoea and headache |
| Page 2020 | Molecular and Cellular Responses to the TYK2/JAK1 Inhibitor PF-06700841 Reveal Reduction of Skin Inflammation in Plaque Psoriasis | NCT02310750 | 1 | PF-06700841 | TYK2/JAK1 | Plaque Psoriasis | All | 18-65 | 30, 100mg QD or placebo for 28 days | 30 | Epidermal thickness and inflammatory cell infiltration; psoriasis-related differentially expressed genes; expression of genes in IL-23/IL-22 and Type1 IFN pathyway | - | - |
| Papp 2018 | Phase 2 Trial of Selective Tyrosine Kinase 2 Inhibition in Psoriasis | NCT02931838 | 2 | BMS-986165 | TYK2 | Plaque Psoriasis | All | 18-70 | 3mg QOD, 3mg QD, 3mg BID, 6mg BID, 12mg QD or placebo (1:1:1:1:1:1) | 267 | 75% or greater reduction from baseline in the PASI score at week 12 | PASI score reductions from baseline of 50% or more, 90% or more, and 100%; sPGA score of 0 or 1 ; QLDI score of 0 or 1 at week 12. | Nasopharyngitis, headache, diarrhea, nausea, upper respiratory tract infection, pruritus, acne, toothache, psoriasis, and aphthous ulcer, malignant melanoma (1 case) |
| Sandbron 2020 | Development of Gut-Selective Pan-Janus Kinase Inhibitor TD-1473 for Ulcerative Colitis: A Translational Medicine Programme | NCT02657122 | 1 | TD-1473 | pan-JAK | Healthy participants | All | 19-55 | SAD: 10, 30, 100, 300 or 1000 mg once, or placebo; MAD: 10, 30, 100 or 300 mg QD, or placebo (3:1) | 72 | Safety | - | - |
|  |  | NCT02818686 | 1b |  |  | Ulcerative Colitis | All | 18-75 | 20, 80, 270mg QD or placebo (1:1:1:1) for 28 days | 40 | Clinical efficacy | Biomarkers | - |
| Singhal 2019 | Oral selective tyrosine kinase 2 (TYK2) inhibition with bms-986165 in patients with systemic lupus erythematosus: A phase 2, randomized, double-blind, placebo-controlled study (Paisley) | NCT03252587 | 2 | BMS-986165 | TYK2 | Systemic Lupus Erythematosus | All | 18-75 | Dose 1, 2, 3 or placebo (1:1:1:1) | 360 | SRI-4 response rate at Week 32 | CLASI response rate, British Isles Lupus Assessment Group-based Composite Lupus Assessment response rate, both at Week 32, and safety | Still ongoing |
| Singhal 2021 | Safety and Pharmacokinetics of the Oral TYK2 Inhibitor PF-06826647: A Phase I, Randomized, Double-Blind, Placebo-Controlled, Dose-Escalation Study | NA | 1 | PF-06826647 | TYK2 | Healthy participants | All | 18-55 | SAD:3, 10, 30, 100, 200, 400, or 1600 mg once or placebo (3:1); MAD: 30, 100, 400, or 1200 mg QD or placebo, 400mg QD or placebo for the Japanese cohort, and 200mg BID or placebo for another separate cohort | 69 | Safety | PK parameters | Constipation, nausea, increased alanine aminotransferase and blood creatinine, headache, and dysuria |
| Tehliran 2021 | Safety, tolerability, efficacy, pharmacokinetics, and pharmacodynamics of the oral TYK2 inhibitor PF-06826647 in participants with plaque psoriasis: a phase 1, randomised, double-blind, placebo-controlled, parallel-group study | NCT03210961 | 1 | PF-06826647 | TYK2 | Plaque Psoriasis | All | 18-55 | Cohort1: 400mg QD or placebo (2:1) for 4w; Cohort2: 100mg QD or placebo (2:1) for 4w. | 40 | Safety of multiple-dose PF-06826647 in participants with plaque psoriasis | Characterisation of the pharmacokinetics of multiple-dose PF-06826647 in plasma and the change in PASI score at day 28 | Constipation, headache, increased blood creatinine, alanine aminotransferase, and aspartate transaminase, decreased lymphocyte |
| Thaci 2022 | Deucravacitinib in Moderate to Severe Psoriasis: Clinical and Quality-of-Life Outcomes in a Phase 2 Trial | NCT02931838 | 2 | BMS-986165 | TYK2 | Plaque Psoriasis | All | 18-70 | 3mg QOD, 3mg QD, 3mg BID, 6mg BID, 12mg QD or placebo (1:1:1:1:1:1) | 267 | Percentages of patients who achieved absolute PASI <= 1, absolute PASI <= 3, absolute PASI <= 5; BSA <= 1%, BSA <= 3%; and >= 75% improvement in sPGA×BSA in 3mg BID, 6mg BID, 12mg QD and placebo group | - | - |
| Winnette 2022 | Characterizing the relationships between patient-reported outcomes and clinician assessments of alopecia areata in a phase 2a randomized trial of ritlecitinib and brepocitinib | NCT02974868 | 2a | PF-06700841 | TYK2/JAK1 | Alopecia Areata | All | 18-75 | 60 mgQD for 4 weeks, then 30 mgQD for 20 weeks or placebo (2:1) | 94 | Change in AASIS scores at week 24; Correlation between SALT scores and AASIS scores at baseline and week 24. | - | - |
| Unpublished1 |  | NCT03895372 | 2b | PF-06826647 | TYK2 | Plaque Psoriasis | All | 18-75 | 16w investigation period: 50, 100, 200, 400 mg QD or placebo, 24w extension period: 200, 400mg QD | 178 | Percentage of participants with a PASI 90 response up to week 16 (investigation period) | Percentage of participants with a PASI 75 response up to week 16 (investigation period) | - |
| Unpublished2 |  | NCT03903822 | 2b | PF-06700841 | TYK2/JAK1 | Atopic Dermatitis | All | 12-75 | 6w intervention: 0.1, 0.3, 1.0, 3.0% cream or placebo (QD), 0.3, 1.0% cream or placebo (BID) | 292 | Percent change from baseline in Eczema Area and Severity Index total score at week 6 | Percentage of participants achieving investigator's global assessment score clear (0) or almost clear (1) and a reduction from baseline of greater than or equal to ( >=2) points at week 6 | - |
| Unpublished3 |  | NCT03715829 | 2b | PF-06700841 | TYK2/JAK1 | Active Non-segmental Vitiligo | All | 18-65 | Dose ranging period: 200mg QD for 4w + 50mg QD for 20w, 100mg QD for 4w+ 50mg QD for 20w; 50, 30, 10 mg QD or placebo for 24w | 364 | Percent change from baseline in Central Read Facial-Vitiligo Area Scoring Index at week 24 | Percentage of participants achieving >= 75% improvement from baseline in Central Read Facial-Vitiligo Area Scoring Index at week 24 | - |

QD, once daily; BID, twice daily; SAD, single-ascending-dose; MAD, multiple-ascending-dose; QOD, every other day; QW, once a week; PASI, Psoriasis Area and Severity Index; sPGA, Static Physician’s Global Assessment; PK, pharmacokinetic; PD, pharmacodynamic; SALT, Severity of Alopecia Tool; ACR-20, American College of Rheumatology-20; PASDAS, Psoriatic Arthritis Disease Activity Score; HAQ-DI, HAQ-Disability Index; SF-36 PCS, Short Form-36 Health Survey Physical Component Summary; FACIT-F, Functional Assessment of Chronic Illness Therapy-Fatigue; QLDI, Dermatology Life Quality Index; SRI-4, Systemic lupus erythematosus Responder Index 4; CLASI, Cutaneous Lupus Erythematosus Disease Area and Severity Index; BSA, body surface area; AASIS, Alopecia Areata Symptom Impact Scale.

**Supplementary Table 15.** Information on 69 randomized controlled trails registered in *clinicaltrail.gov* database

| **NCT number** | **Start Date** | **Sponsor/Collaborators** | **Status** | **Conditions** | **Interventions** | **Target** | **Gender** | **Age** | **Phase** | **N** |
| --- | --- | --- | --- | --- | --- | --- | --- | --- | --- | --- |
| NCT05065762 | 24-Sep-21 | Bristol-Myers Squibb | Not yet recruiting | Psoriasis | BMS-986165 | TYK2 | All | >=20 | - | 310 |
| NCT04949269 | 20-Jul-21 | Bristol-Myers Squibb | Completed | Healthy participants | BMS-986165, Famotidine | TYK2 | All | 18-55 | 1 | 61 |
| NCT04908189 | 15-Jul-21 | Bristol-Myers Squibb | Recruiting | Psoriatic Arthritis | BMS-986165, Placebo, Apremilast | TYK2 | All | >=18 | 3 | 700 |
| NCT04908202 | 13-Jul-21 | Bristol-Myers Squibb | Recruiting | Psoriatic Arthritis | BMS-986165, Placebo | TYK2 | All | >=18 | 3 | 650 |
| NCT04857034 | 12-Jul-21 | Bristol-Myers Squibb | Recruiting | Discoid Lupus Erythematosus, Subacute Cutaneous Lupus Erythematosus | BMS-986165, Placebo | TYK2 | All | 18-75 | 2 | 75 |
| NCT05076006 | 21-May-21 | Emma Guttman, Pfizer, Icahn School of Medicine at Mount Sinai | Recruiting | Cicatricial Alopecia | PF-06700841, Placebo | TYK2/JAK1 | All | >=18 | 2 | 48 |
| NCT04877990 | 07-May-21 | Bristol-Myers Squibb | Recruiting | Crohn Disease, Ulcerative Colitis | BMS-986165 | TYK2 | All | >=18 | 2 | 300 |
| NCT04772079 | 23-Mar-21 | Bristol-Myers Squibb | Recruiting | Plaque Psoriasis | BMS-986165, Placebo | TYK2 | All | 12-18 | 3 | 84 |
| NCT04613518 | 15-Mar-21 | Bristol-Myers Squibb | Recruiting | Ulcerative Colitis | BMS-986165, Placebo | TYK2 | All | 18-65 | 2 | 50 |
| NCT04671953 | 18-Dec-20 | Bristol-Myers Squibb | Completed | Healthy participants | BMS-986165, Metformin | TYK2 | All | 18-50 | 1 | 36 |
| NCT04591262 | 10-Nov-20 | Pfizer | Completed | Healthy participants | PF-06826647 | TYK2 | Male | 18-55 | 1 | 6 |
| NCT04587713 | 16-Oct-20 | Theravance Biopharma | Completed | Intestinal Disorders, Inflammatory Bowel Diseases | TD-1473 | Pan-JAK | All | 19-55 | 1 | 58 |
| NCT04580797 | 02-Oct-20 | Pfizer | Completed | Healthy participants | PF-06700841, Placebo | TYK2/JAK1 | All | 18-55 | 1 | 36 |
| NCT04209556 | 30-Sep-20 | Pfizer | Withdrawn | Ulcerative Colitis | PF-06826647, Placebo | TYK2 | All | 18-75 | 2 | 0 |
| NCT04353791 | 16-Sep-20 | Oncostellae S.L | Recruiting | Ulcerative Colitis | OST-122, Placebo | TYK2/JAK3/ARK5 | All | 18-75 | 1, 2 | 32 |
| NCT04536961 | 10-Sep-20 | Bristol-Myers Squibb | Completed | Healthy participants | BMS-986165, Famotidine | TYK2 | All | 18-55 | 1 | 56 |
| NCT04305899 | 31-Aug-20 | Bristol-Myers Squibb | Completed | Healthy participants | BMS-986165 | TYK2 | All | 18-55 | 1 | 40 |
| NCT04267250 | 24-Aug-20 | Pfizer | Completed | Healthy participants | PF-06700841, Ethinyl estradiol (EE) and levonorgestrel (LN) | TYK2/JAK1 | Female | 18-60 | 1 | 18 |
| NCT03920254 | 23-Jul-20 | Theravance Biopharma | Terminated | Ulcerative Colitis | TD-1473 | Pan-JAK | All | >=18 | 2, 3 | 46 |
| NCT04260464 | 03-Jul-20 | Pfizer | Recruiting | Healthy participants, Renal Impairment | PF-06700841 | TYK2/JAK1 | All | 18-75 | 1 | 32 |
| NCT04249284 | 06-Feb-20 | Bristol-Myers Squibb | Completed | Healthy participants | BMS-986165 | TYK2 | All | 18-45 | 1 | 18 |
| NCT04209699 | 27-Dec-19 | Bristol-Myers Squibb | Completed | Healthy participants | BMS-986165, Famotidine | TYK2 | All | 18-55 | 1 | 18 |
| NCT04092452 | 02-Dec-19 | Pfizer | Completed | Acne Inversa | PF-06826647, PF-06700841, PF-06650833, Placebo | PF-06826647 (TYK2), PF-06700841 (TYK2/JAK1) | All | 18-75 | 2 | 197 |
| NCT04167462 | 25-Nov-19 | Bristol-Myers Squibb | Completed | Psoriasis | BMS-986165, Placebo | TYK2 | All | >=18 | 3 | 220 |
| NCT04175925 | 14-Nov-19 | Bristol-Myers Squibb | Active, not recruiting | Healthy participants | BMS-986322, Placebo, famotidine | TYK2 | All | 18-60 | 1 | 432 |
| NCT04134715 | 23-Oct-19 | Pfizer | Completed | Healthy participants | PF-06826647, Oral Contraceptive | TYK2 | Female | 18-60 | 1 | 15 |
| NCT04113668 | 01-Oct-19 | Bristol-Myers Squibb | Completed | Healthy participants | BMS-986165, diflunisal | TYK2 | All | 18-50 | 1 | 48 |
| NCT04090047 | 24-Sep-19 | Pfizer | Completed | Healthy participants | PF-06700841, Itraconazole | TYK2/JAK1 | All | 18-55 | 1 | 12 |
| NCT04086719 | 12-Sep-19 | Bristol-Myers Squibb | Completed | Healthy participants | BMS-986165, Pyrimethamine | TYK2 | Male | 18-50 | 1 | 60 |
| NCT04065932 | 22-Aug-19 | Bristol-Myers Squibb | Completed | Lupus | BMS-986165, Famotidine | TYK2 | All | 18-50 | 1 | 33 |
| NCT04055506 | 14-Aug-19 | Bristol-Myers Squibb | Completed | Healthy participants | BMS-986165, Ritonavir | TYK2 | All | 18-50 | 1 | 16 |
| NCT04036435 | 12-Aug-19 | Bristol-Myers Squibb | Active, not recruiting | Psoriasis | BMS-986165 | TYK2 | All | >=18 | 3 | 1452 |
| NCT03943147 | 15-Jul-19 | Bristol-Myers Squibb | Terminated | Lupus Nephritis | BMS-986165, Placebo, Mycophenolate Mofetil | TYK2 | All | 18-75 | 2 | 1 |
| NCT04254549 | 14-Jun-19 | Mayo Clinic | Recruiting | Crohn Disease, Diabetic Gastroparesis | TD-1473, Rifaximin, Placebo | Pan-JAK | All | 18-75 | 2 | 40 |
| NCT03979248 | 16-May-19 | Bristol-Myers Squibb | Completed | Healthy participants | BMS-986165, Rabeprazole | TYK2 | All | 18-50 | 1 | 21 |
| NCT03930602 | 01-May-19 | Bristol-Myers Squibb | Completed | Healthy participants | BMS-986165, Fluvoxamine | TYK2 | All | 18-50 | 1 | 16 |
| NCT03845517 | 18-Apr-19 | Pfizer | Recruiting | Systemic Lupus Erythematosus | PF-06700841, Placebo | TYK2/JAK1 | All | 18-75 | 2 | 448 |
| NCT03924427 | 10-Apr-19 | Bristol-Myers Squibb | Completed | Psoriasis | BMS-986165 | TYK2 | All | >=20 | 3 | 74 |
| NCT03850483 | 08-Apr-19 | Pfizer | Completed | Psoriasis | PF-06700841, Vehicle | TYK2/JAK1 | All | 18-75 | 2 | 344 |
| NCT03956953 | 04-Apr-19 | Bristol-Myers Squibb | Completed | Autoimmune Diseases | BMS-986165, Placebo | TYK2 | All | 18-45 | 1 | 135 |
| NCT03890770 | 04-Apr-19 | Bristol-Myers Squibb | Completed | Renal Impairment, Healthy participants | BMS-986165 | TYK2 | All | 18-70 | 1 | 44 |
| NCT03920267 | 26-Mar-19 | Bristol-Myers Squibb | Active, not recruiting | Systemic Lupus Erythematosus | BMS-986165 | TYK2 | All | 18-75 | 2 | 261 |
| NCT03916250 | 23-Mar-19 | Pfizer | Completed | Healthy participants | PF-06700841, White petrolatum | TYK2/JAK1 | All | 20-55 | 1 | 20 |
| NCT03758443 | 11-Mar-19 | Theravance Biopharma | Terminated | Ulcerative Colitis | TD-1473, Placebo | Pan-JAK | All | >=18 | 2, 3 | 243 |
| NCT03890809 | 26-Feb-19 | Bristol-Myers Squibb | Completed | Liver Dysfunction, Healthy participants | BMS-986165 | TYK2 | All | 18-70 | 1 | 32 |
| NCT03873415 | 25-Jan-19 | Bristol-Myers Squibb | Completed | Psoriasis | BMS-986165 | TYK2 | Male | 18-45 | 1 | 9 |
| NCT03765554 | 07-Jan-19 | Pfizer | Completed | Healthy participants | PF-06700841 | TYK2/JAK1 | All | 18-55 | 1 | 8 |
| NCT03770039 | 10-Dec-18 | Pfizer | Completed | Healthy participants | PF-06700841 | TYK2/JAK1 | Male | 18-55 | 1 | 6 |
| NCT03739788 | 05-Dec-18 | Bristol-Myers Squibb | Completed | Healthy participants | BMS-986165 | TYK2 | Male | 18-55 | 1 | 8 |
| NCT03750565 | 27-Nov-18 | Theravance Biopharma | Completed | Inflammatory Bowel Diseases | TD-1473, Placebo | Pan-JAK | All | 18-55 | 1 | 60 |
| NCT03635112 | 19-Nov-18 | Theravance Biopharma | Terminated | Crohn's Disease | TD-1473, Placebo | Pan-JAK | All | >=18 | 2 | 167 |
| NCT03751228 | 11-Oct-18 | Bristol-Myers Squibb | Completed | Healthy participants | BMS-986165, Active Pharmaceutical Ingredient | TYK2 | All | 25=80 | 1 | 9 |
| NCT03656952 | 05-Sep-18 | Pfizer | Completed | Healthy participants | PF-06700841, Placebo, moxifloxacin | TYK2/JAK1 | All | 18-55 | 1 | 33 |
| NCT03660436 | 14-Aug-18 | Bristol-Myers Squibb | Completed | Healthy participants | BMS-986165, MMF | TYK2 | Male | 18-50 | 1 | 131 |
| NCT03611751 | 26-Jul-18 | Bristol-Myers Squibb | Completed | Psoriasis | BMS-986165, Placebo, Apremilast | TYK2 | All | >=18 | 3 | 1020 |
| NCT03599622 | 16-Jul-18 | Bristol-Myers Squibb | Recruiting | Granulomatous Colitis, Crohn's Disease, Crohn's Enteritis, Granulomatous Enteritis | BMS-986165, Placebo | TYK2 | All | 18-75 | 2 | 240 |
| NCT03555617 | 13-Jun-18 | Theravance Biopharma | Completed | Inflammatory Bowel Diseases | TD-1473, Itraconazole | Pan-JAK | All | 19-55 | 1 | 36 |
| NCT03541564 | 30-May-18 | Bristol-Myers Squibb | Completed | Systemic Lupus Erythematosus, Healthy participants | BMS-986165, Moxifloxacin, Placebo | TYK2 | All | 18-50 | 1 | 84 |
| NCT03419910 | 05-Mar-18 | Bristol-Myers Squibb | Completed | Psoriasis | BMS-986165, Cyclosporine | TYK2 | Male | 18-50 | 1 | 54 |
| NCT03395184 | 02-Feb-18 | Pfizer | Recruiting | Crohn's Disease | PF-06700841, Placebo | TYK2/JAK1 | All | 18-75 | 2 | 250 |
| NCT03408470 | 31-Jan-18 | Theravance Biopharma | Completed | Intestinal Disorders, Inflammatory Bowel Diseases | TD-1473 | Pan-JAK | Male | 19-55 | 1 | 20 |
| NCT03402087 | 08-Jan-18 | Bristol-Myers Squibb | Completed | Lupus, Psoriatic Arthritis | BMS-986165, Methotrexate, Leucovorin | TYK2 | Male | 18-50 | 1 | 30 |
| NCT03254784 | 13-Sep-17 | Bristol-Myers Squibb | Completed | Systemic Lupus Erythematosus, Arthritic Psoriasis, Psoriasis, Inflammatory Bowel Diseases | BMS-986165 | TYK2 | All | 18-50 | 1 | 49 |
| NCT03262727 | 01-Sep-17 | Bristol-Myers Squibb | Completed | Systemic Lupus Erythematosus, Arthritic Psoriasis, Psoriasis, Inflammatory Bowel Diseases | BMS-986165, Loestrin | TYK2 | Female | 18-40 | 1 | 49 |
| NCT03236493 | 16-Aug-17 | Pfizer | Completed | Healthy participants | PF-06700841, Placebo | TYK2/JAK1 | All | 18-55 | 1 | 8 |
| NCT02958865 | 03-Feb-17 | Pfizer | Completed | Ulcerative Colitis | PF-06700841, Placebo | TYK2/JAK1 | All | 18-75 | 2 | 319 |
| NCT03044873 | 02-Feb-17 | Bristol-Myers Squibb | Completed | Autoimmune Diseases, Inflammatory Diseases | BMS-986165, Rosuvastatin | TYK2 | All | 18-50 | 1 | 20 |
| NCT03004768 | 26-Jan-17 | Bristol-Myers Squibb | Completed | Psoriasis | BMS-986165 | TYK2 | Male | 18-50 | 1 | 6 |
| NCT02534636 | Oct-15 | Bristol-Myers Squibb | Completed | Healthy participants | BMS-986165, Interferon alpha-2a recombinant, Famotidine, Placebo | TYK2 | All | 18-70 | 1 | 140 |

**
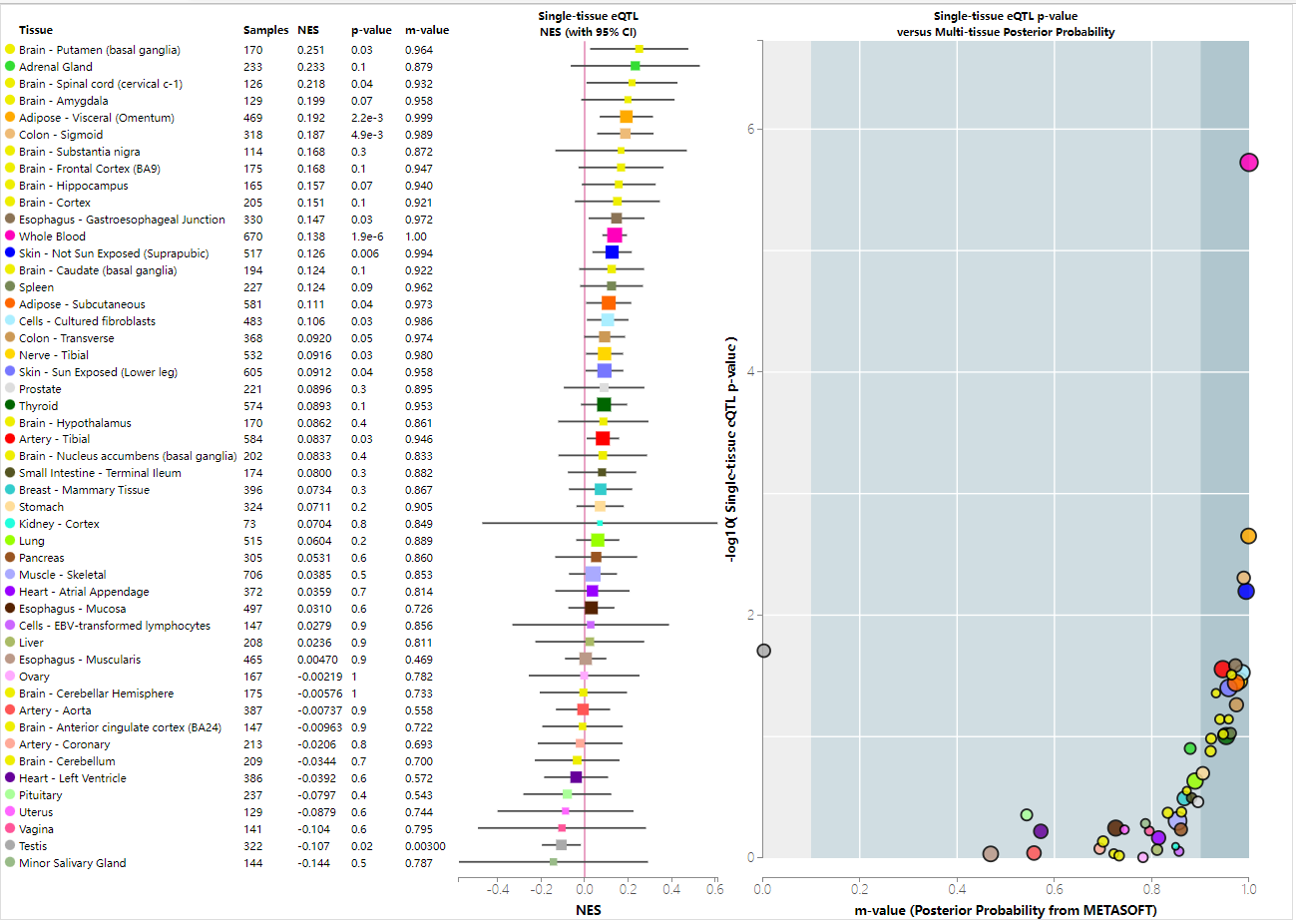
**

**Supplementary Figure 1.** Rs34536443 in *TYK2* gene region differently expressed across 45 tissues

**
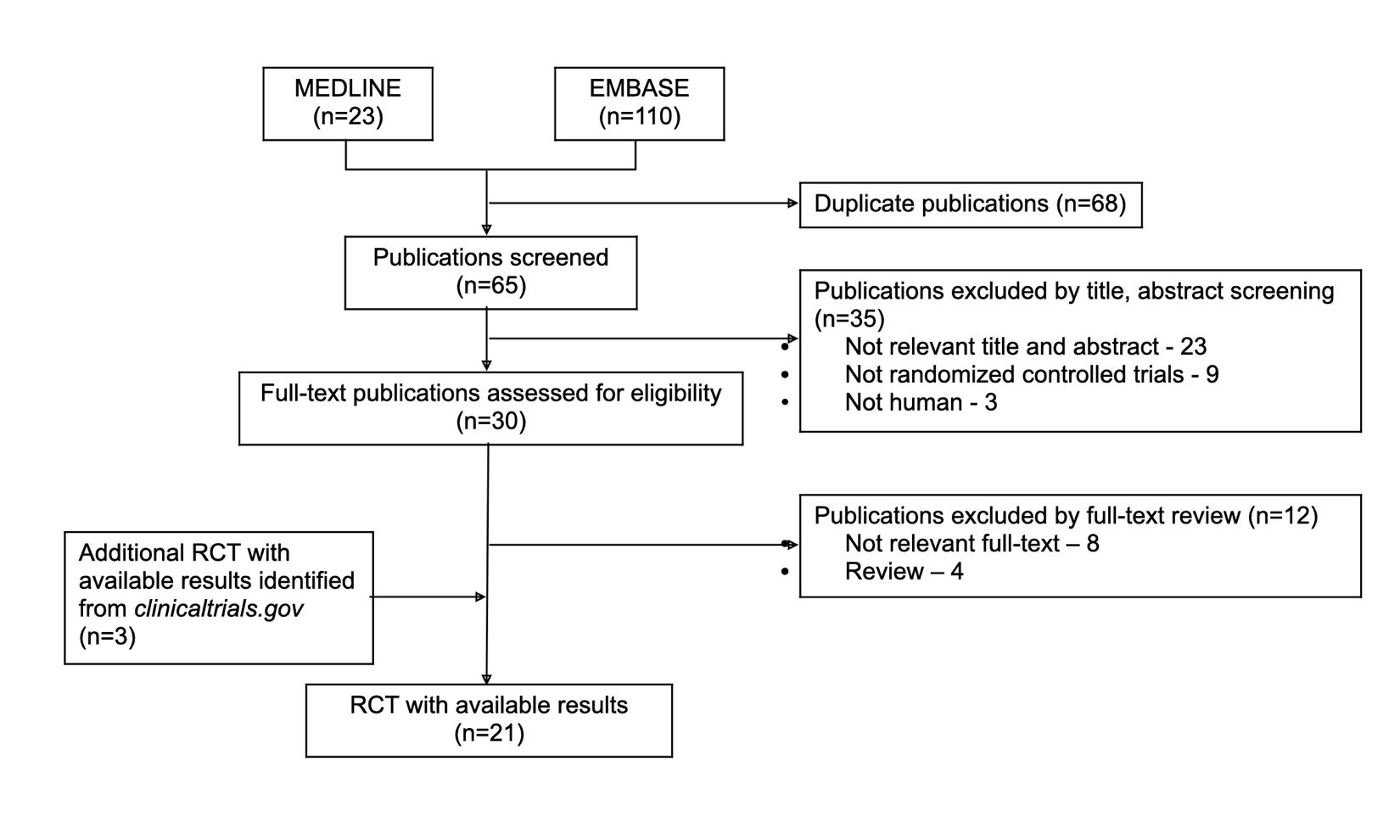
**

**Supplementary Figure 2.** Flow chart of study selection for systematic review on trials on TYK2 inhibitor

**References**:

1. Astle WJ, Elding H, Jiang T, Allen D, Ruklisa D, Mann AL, et al. The Allelic Landscape of Human Blood Cell Trait Variation and Links to Common Complex Disease. Cell. 2016 Nov 17; 167(5):1415-1429 e1419.

2. Ahola-Olli AV, Wurtz P, Havulinna AS, Aalto K, Pitkanen N, Lehtimaki T, et al. Genome-wide Association Study Identifies 27 Loci Influencing Concentrations of Circulating Cytokines and Growth Factors. Am J Hum Genet. 2017 Jan 5; 100(1):40-50.

3. Scott RA, Lagou V, Welch RP, Wheeler E, Montasser ME, Luan J, et al. Large-scale association analyses identify new loci influencing glycemic traits and provide insight into the underlying biological pathways. Nat Genet. 2012 Sep; 44(9):991-1005.

4. Kettunen J, Demirkan A, Wurtz P, Draisma HH, Haller T, Rawal R, et al. Genome-wide study for circulating metabolites identifies 62 loci and reveals novel systemic effects of LPA. Nat Commun. 2016 Mar 23; 7:11122.

5. Orrù V, Steri M, Sidore C, Marongiu M, Serra V, Olla S, et al. Complex genetic signatures in immune cells underlie autoimmunity and inform therapy. Nat Genet. 2020 Oct; 52(10):1036-1045. doi: 1010.1038/s41588-41020-40684-41584. Epub 42020 Sep 41514.
